# Exploratory analysis of the ecological variables associated with sexual health profiles in high-risk, sexually-active female learners in rural KwaZulu-Natal

**DOI:** 10.1371/journal.pone.0195107

**Published:** 2018-04-05

**Authors:** Hilton Humphries, Farzana Osman, Lucia Knight, Quarraisha Abdool Karim

**Affiliations:** 1 Centre for the AIDS Programme of Research in South Africa (CAPRISA), Nelson R Mandela School of Medicine, University of KwaZulu-Natal, Durban, South Africa; 2 School of Public Health, University of the Western Cape, Cape Town, South Africa; 3 Department of Epidemiology, Mailman School of Public Health, Columbia University, New York City, United States of America; Massachusetts General Hospital, UNITED STATES

## Abstract

**Purpose:**

Young women are at high risk for negative sexual health outcomes. Despite their high risk, many sexually-active women never experience negative sexual health outcomes. This study explored the ecological risk factors associated with the risk profiles of sexually-active female high school-learners in rural KwaZulu-Natal, South Africa.

**Methods:**

Using baseline data from N = 596 sexually-active school-going women, we explored the ecological factors associated with being sexually-active and managing risk successfully [SARS] or unsuccessfully [SARU]. Generalised estimated equations (GEE) were applied to data collected at multiple levels while adjusting for school and other included variables. GEE were used to calculate probability of being SARU.

**Results:**

Amongst SARU learners, 21.9% had HIV, 38.6% had HSV-2, 12.5% were pregnant, 28.7% self-reported STI symptoms and 51.9% reported a previous pregnancy. Individual-level factors had the greatest impact on being SARU. Univariate and multivariate analysis highlighted several important partner factors associated with SARU. Age was significantly associated with the risk profiles (p<0.0001), a greater proportion of SARU learners were 18 or older compared to the SARS learners. The odds of being SARU decreased when ≥18 years (aOR = 0.2577, 95% CI 0.1462–0.4542) or if not falling pregnant was important (aOR = 0.6343, 95% CI 0.4218–0.9538). Having >1 HIV test (aOR = 2.2161, 95% CI 1.3964–3.5169) increased the odds a SARU profile.

**Conclusion:**

Individual and partner level factors are important for the sexual health profile of an adolescent female. While the exploratory findings require further research; managing multiple sexual health outcomes, tailoring responses around a risk profile and including partners is essential for successful interventions.

## Introduction

Adolescents are at high risk of experiencing negative sexual health outcomes (HIV infection, STI infection and early pregnancy)[1,2]. The prevalence of negative sexual health outcomes is unacceptably high, with young women bearing the highest burden[2–5]. In sub-Saharan Africa, young women aged 15–24 years accounted for 25% of new HIV infections amongst adults in 2015[4],HSV-2 prevalence may be as high as 53% amongst young women aged 13–24 years[6,7] and account for a significant proportion of the approximately 16 million girls aged 15–19 that give birth every year[8]. In South Africa, school-going women aged 13–24 years already have a 6% HIV prevalence, 10.7% HSV-2 prevalence and 3.6% pregnancy prevalence[9].

Essential to increased vulnerability of negative sexual reproductive health (SRH) outcomes and HIV infection is being sexually active[3,10]. Recent data suggests that 10–20% of young people aged 15–24 years old in sub-Saharan Africa are sexually active and therefore potentially at risk of a negative sexual outcome[1]. Despite the high-risk context of South Africa, only some sexually active young women experience negative SRH outcomes. When assessing high-risk, research has focused on a single SRH outcome (such as HIV) or on certain key behaviours (such as condom use)[11]. There has been less investigation into the differences between sexually active young women, and why despite similar high-risk settings, some manage to avoid negative SRH outcomes while others do not. An understanding of why some young women manage their SRH risk better than others is critical for designing contextualised and localised prevention interventions that better target high-risk adolescents.

Defining risk profiles could provide a useful tool for conceptualising risk in adolescents. Focusing on sexually active adolescents, we identify specific risk categories, including those who are: 1) sexually active and have experienced negative SRH outcomes (SARU), and those 2) sexually active and have never experienced negative SRH (SARS) despite a similar high-risk context. Research suggests that the factors that may affect adolescent SRH outcomes are complex and ecologically diverse[1,3,11,12]. Understanding how individual factors [2,12–18], peer groups[19–22],partners[20,22–24],familial context[25–27], the relationship, quality and exposure to school[21,24,28–30], and broader cultural and national factors[31–36] contribute to the difference in risk of these profiles is important to inform programmes and policies.

We used baseline data from a cluster-randomised control trial[9,37] to explore the ecological differences between sexually-active female learners who had experienced a negative health outcome and those that had not despite living in a similar high-risk context.

## Materials and methods

### Study setting and population

We analysed baseline data from a cluster randomised control trial undertaken between 2010 and 2013. The study took place in 14 schools in Vulindlela, a rural sub-district in KwaZulu-Natal, South Africa. The schools were selected from 42 secondary schools in the district, based on size, access, and school pass rates. All learners in grades 9 and 10, were eligible for inclusion. Details of the study are provided elsewhere[9,37]. All participants provided written informed consent prior to being enrolled. Learners ≥18 years provided first person consent following a literacy and comprehension assessment, learners <18 years, provided assent, while written consent was obtained from the parent/guardian or parental proxy where necessary. All ethical approvals were granted by the University of KwaZulu-Natal Biomedical Ethics committee (BF105/010 and BE 523/14).

### Data collection

For the main study, behavioural and demographic data were collected using self-completed, structured questionnaires available in *isiZulu* and English. Biological measures included HIV, HSV-2, and urine pregnancy testing (details of which tests were used to measure biological outcomes in the main study are presented elsewhere[9]). Respondents with a positive result on biological tests, or potential symptoms of STI infection, or requiring support for experiencing forced sex were referred as necessary to local services.

### Study variables

For this secondary analysis, we explored the relationship between ecological risk factors and the risk profiles of sexually active adolescent women. The dependant variable was the risk profiles of the sexually-active young women, it involved the experience of ≥1 negative sexual health outcome; HIV infection, HSV-2 infection, positive pregnancy, self-reported previous pregnancy and STI symptoms. Sexual activity was based on self-report of having ever had sex, except where the participant reported not being sexually active but was positive for at-least one of the SRH outcomes in which case they were considered sexually active. Using our definition, we identified 148 (24.8%) additional female learners, increasing the number of sexually-active learners from the main study[9]. We investigated the differences between two risk profiles, defined for the purpose of this study as:

- **Sexually active and managed risk unsuccessfully (SARU):** A participant was coded into this risk profile if they were sexually active, and had one or more negative sexual health outcome.
- **Sexually active and managed risk successfully (SARS):** A participant was coded into this risk profile if they reported being sexually active but had not experienced a negative sexual health outcome.

An ecological framework was used to guide the analysis of the independent variables, by providing a theoretical framework for visualising and understanding the complexity of variables that influence the risk of an individual for negative sexual health outcomes[38]. Ecological variables included were chosen from a review of the literature on risk factors associated with increased risk of negative SRH outcomes. These variables come from multiple ecological levels including individual, family, and partner/peer, school, health-care and community level variables and are outlined in S1 Table.

### Analysis

As young women are at highest risk of multiple SRH outcomes[1,3,9], the current analysis focused on sexually-active female learners. The demographic, behavioural and biological characteristics were summarised using descriptive summary statistics (Table 1). It was necessary to adjust for any cluster effects arising from the school-based sampling. Similar to previous analyses[9], cluster-level summaries were computed. In the adjusted analysis prevalence for each school (cluster) was calculated and then averaged. An unadjusted analysis was also completed, however this analysis did not consider the clustering and calculated prevalence by combining prevalence of all clusters. To compare the two dependant variable categories, we used a t-test for two independent samples using the adjusted prevalence estimates from each of the 14 clusters.

**Table 1:** Demographics and basic behavioural characteristics of sexually active students in rural KwaZulu-Natal, South Africa.

Basic Demographics Female							
Variable		Female Overall	Female SARU		Female SARS		p-value
		unadjusted % (n/N)	unadjusted % (n/N)	adjusted %*	unadjusted % (n/N)	adjusted %*	
		N = 596	N = 395		N = 201		
**Age Median(IQR)**		17yo(16–18)	17yo(16–19)		16yo(15–17)		**<0.000**
**Age Category**		N = 596	66(N = 395)		33.7(N = 201)		
	**< = 15**	24.2(144)	22.0(87)	23.6	28.4(57)	28.9	**<0.000**
	**16–17**	40.3(240)	33.9(134)	35.4	52.7(106)	52.7	
	**18–19**	23.8(142)	27.8(110)	26.8	15.9(32)	14.5	
	**> = 20**	11.7(70)	16.20(64)	14.2	3.0(6)	3.9	
**Head of household (HoH)**		N = 594	66.2(N = 393)		33.8(N = 201)		
	**Both Parents**	35(208)	34.4(135)	33.5	36.3(73)	36.2	0.939
	**Birth Mother**	13.3(79)	13.5(53)	13.0	12.9(26)	11.4	
	**Birth father**	15.3(91)	16.0(63)	17.1	13.4(28)	12.6	
	**Grandparent**	1.3(8)	1.27(5)	1.2	1.5(3)	1.5	
	**Child headed**	18.0(107)	18.8(74)	19.0	16.4(33)	17.9	
	**Other**	2.0(12)	1.8(7)	1.6	2.5(5)	3.0	
	**Sibling older than 18**	15.0(89)	14.3(56)	14.6	16.4(33)	17.4	
**Adult Death**		N = 591	66(N = 390)		34(N = 201)		
	**0**	45.9(271)	45.38(177)	46.4	46.8(94)	48.0	0.898
	**1**	20,0(118)	20.51(80)	20.1	18.9(38)	19.3	
	**>1**	34.2(202)	34.10(133)	33.4	34.3(69)	32.7	

*Adjusted analysis accounts for cluster effects arising from the school-based sampling

A univariate and multivariate analysis was conducted in order to identify variables associated with the two key risk profiles in the 596 young women identified as sexually-active. These analyses were completed using generalised estimating equations. This method was used to calculate the adjusted Odds Ratios (aOR) with 95% Confidence Intervals (CIs) modelling the probability of being sexually active and experiencing a negative SRH outcome. The self-completed nature of the data collection, meant that there was missing data on certain variables. In the multivariate analysis, we included variables identified in the univariate with a p-value<0.2, but variables missing >20% of the responses were excluded. We tested for multicollinearity using the Variance Inflation Factor (VIF) criteria. If variables were highly correlated, we excluded them from the model. The analysis was unweighted, and we did not adjust for non-response. As the outcome variable was determined by self-reported sexual activity, except where the participant reported not being sexually active but was positive for at-least one of the SRH outcomes, we assumed that the data was missing at random and was not related to the outcome variable. SAS statistical package (V.9.4; Statistical Analysis Software, North Carolina, USA) and IBM SPSS (Version 24) were used for the analysis.

## Results

### Young women in the SARU and SARS risk profile

At baseline, there were a total of N = 1423 grade 9 and 10 female learners who consented for inclusion from 14 schools enrolled into the study[9,39]. Using our definition, we identified 596 sexually active young women, 66.3% of which were SARU and 33.7% SARS for inclusion in the current analyses. The prevalence of negative health outcomes highlighted the high-risk of sexually active learners, overall, HIV prevalence was 14.8% (88/596), HSV-2 prevalence was 24.8% (148.596), STI symptoms 18.6% (110/592), pregnancy 8.3% (49/592). Amongst those who had experienced a negative sexual health outcome, 21.9% (88/395) had HIV, 38.6% (148/395) had HSV-2, 12.5% (49/391) were pregnant, 28.7% (110/394) had self-reported STI symptoms and 51.9% (147/283) reported a previous pregnancy (S2 Table).

### Demographics and sexual behaviour variables

The results of the descriptive analysis are reported in Table 1. Overall the median age of those identified as sexually active was 17 (IQR16-18) years, with a median age of 17 (IQR16-19) years for SARU learners and 16 (IQR15-17) years for SARS learners. Overall, SARS learners had a median age that was significantly younger than SARU learners (p<0.0001). Age was significantly associated with the risk profiles (p<0.0001), a greater proportion of SARU learners were 18 or older (41%) compared to the SARS learners where most learners were under 18 years old (81.6%). While head of household was not significantly associated with the profiles, most learners in both categories self-reported that their mother was the head of the household (34.4% in SARU, 36.3% in SARS).

Key sexual behaviours showed no significant differences between the profiles except for the experience of oral sex where SARS learners were more likely to have experienced oral sex (p = 0.0053) than SARU learners. Young women experience high rates of sexual violence, although not associated with the profiles, 16.2% and 17.1% of SARU and SARS respectively had experienced a threat of violence for sex (S2 Table).

### Univariate analysis: Risk factors associated with a negative sexual health outcome

Table 2 presents the univariate and multivariate analyses of the ecological behavioural factors associated with being SARU compared to those that are SARS. Table 2 includes variables with a p value <0.2 at univariate analysis; other variables are included in the supplementary data (S3 Table) (Note that univariate variables discussed in this section that are not included in Table 2 can be found in the S3 Table). At an individual ecological level, many of the variables that increased the odds of a female learner being SARU in the unadjusted analysis were related to sexual behaviours. Female learners who had oral sex when they were younger than 18 years (OR = 3.429, 95% CI 2.248–5.231), had experienced vaginal sex ever/or vaginal sex at their last sex act (OR = 4.068, 95% CI 1.95–8.488; OR = 3.609, 95% CI 1.89–6.891), had sex which included a combination of oral, vaginal and anal sex ever/or at their last sex act (OR = 4.392, 95% CI 2.004–9.623; OR = 2.044, 95% CI 1.086–3.847), had one or more partners (OR = 1.681, 95% CI 1.064–2.658, OR = 2.319, 95% CI 1.43–3.76), had transactional sex (OR = 1.509, 95% CI 0.965–2.36) or a new partner in the last 12 months (OR = 1.805, 95% CI 1.063–3.066), all had a higher odds of being SARU than SARS. In addition to variables related to sexual activity, female learners who had previously had an HIV test (OR = 2.256, 95% CI 1.712 to 2.971) had increased odds of being SARU. The only variable that decreased the odds of being SARU for learners at an individual ecological level was the current use of contraception (OR = 0.561, 95% CI 0.287–1.096).

**Table 2:** Univariate and multivariate analysis of factors associated with SARU vs SARS profiles amongst learners in rural KwaZulu-Natal, South Africa^@^.

			Female Univariate Analysis				Female Multivariate Analysis			
	
		% (n/N)	Odd Ratio	CI (95%)		Sig.	Odd Ratio	CI (95%)		p-value
Characteristic				Lower	Upper			Lower	Upper	
**Individual Level Factors**										
**Age**	**Under 18**	64.4 (384/596)	**1.00 (Ref)**				**1.00(ref)**			
** **	**18 or older**	35.6 (212/596)	**3.429**	**2.248**	**5.231**	**<0.001**	**0.258**	**0.146**	**0.454**	**<0.000**
**Alcohol Use**	**No**	78.9% (463/587)	1.00 (Ref)				1.00(ref)			
** **	**Yes**	21.1% (124/587)	0.778	0.531	1.139	0.196	0.764	0.502	1.163	0.210
**Self-efficacy**	**Low**	31.4 (187/596)	1.00 (Ref)				1.00(ref)			
** **	**Medium**	53.4 (318/596)	1.308	0.903	1.895	0.156	0.688	0.359	1.320	0.261
** **	**High**	15.1 (90/596)	1.538	0.903	2.621	0.113	0.884	0.478	1.636	0.694
**Correct beliefs on HIV**	**Low**	12.1% (72/594)	**1.00 (Ref)**				**1.00(ref)**			
** **	**Medium**	64.5% (383/594)	**1.678**	**1.052**	**2.677**	**0.03**	**1.112**	0.566	2.187	0.758
** **	**High**	23.4% (139/594)	1.616	0.905	2.884	0.105	0.975	0.429	2.217	0.952
**Accuracy of key condom knowledge**	**Low**	19.0 (113/596)	**1.00 (Ref)**				**1.00(ref)**			
** **	**Medium**	28.5 (169/596)	**1.901**	**1.475**	**2.450**	**0.000**	**1.487**	**1.085**	**2.038**	**0.014**
** **	**High**	52.5 (312/596)	**1.433**	**1.028**	**1.999**	**0.034**	1.183	0.811	1.723	0.382
**Importance of not falling pregnant at school**	**Not Important**	30% (176/586)	**1.00 (Ref)**				**1.00(ref)**			
** **	**Important**	70% (410/586)	**0.763**	0.548	1.062	0.109	**0.634**	**0.422**	**0.954**	**0.029**
**Contraception responsibility**	**not important**	7.2% (42/584)	**1.00 (Ref)**				**1.00(ref)**			
** **	**female**	37.8% (221/584)	**2.215**	0.879	5.584	0.092	**2.743**	**1.209**	**6.225**	**0.016**
** **	**male**	3.1% (18/584)	**0.958**	0.481	1.909	0.903	0.878	0.220	3.499	0.854
** **	**both partners**	43.2% (252/584)	**1.86**	**1.087**	**3.186**	**0.024**	**2.435**	**1.487**	**3.985**	**0.000**
** **	**other**	8.7% (51/584)	**1.511**	0.694	3.291	0.298	2.281	0.858	6.065	0.098
**Number of HIV tests**	**0**	46.4% (265/571)	**1.00 (Ref)**				1.00(ref)			
** **	**1**	16.1% (92/571)	**1.436**	0.946	2.18	0.09	1.374	0.875	2.157	0.167
** **	**>1**	37.5% (214/571)	**2.825**	**1.917**	**4.164**	**<0.000**	**2.216**	**1.396**	**3.517**	**0.001**
**Repeated a grade**	**No**	46.3% (271/585)	**1.00 (Ref)**				1.00(ref)			
	**Yes**	53.7% (314/585)	**1.564**	**1.026**	**2.385**	**0.038**	0.736	0.394	1.372	0.334
**Partner/Peer Level factors**										
**Pressure to have sex (peer)**	**No**	79.3% (471/594)	**1.00 (Ref)**				1.00(ref)			
** **	**Yes**	20.7% (123/594)	**0.627**	**0.395**	**0.996**	**0.048**	0.564	0.275	1.157	0.118
**Community Level factors**										
**Women can have equal say in relationships**	**No**	40.2% (236/587)	**1.00 (Ref)**				1.00(ref)	0.130	0.842	
** **	**Yes**	59.8% (351/587)	**1.495**	**1.044**	**2.141**	**0.028**	1.427	0.878	2.320	0.151
**HIV AIDS important community issues**	**No**	44% (260/591)	**1.00 (Ref)**				1.00(ref)			
** **	**Yes**	56% (331/591)	0.868	0.711	1.059	0.163	1.024	0.754	1.390	0.881
**Social Activity Participation**	**No**	91.9 (546/594)	1.00 (Ref)				1.00(ref)			
	**Yes**	8.1 (48/594)	0.623	0.308	1.258	0.187	0.631	0.245	1.624	0.340

^@^ Note that adjusted analysis adjusted for variables included in the multivariate model.

At a peer/partner level, having a partner who was over four years older was associated with a three-fold increase in the odds of having a negative sexual health outcome (OR = 3.046, 95% CI 1.534–6.048 and OR = 5.459, 95% CI 1.788–16.667), while those who knew their partner’s status had a two-fold increased chance of being SARU (OR = 2.134, 95% CI 1.233–3.694) than SARS. There were no factors associated with an increased or decreased odds of being SARU at the family and school ecological level. At a community level, the belief that women should have an equal say in relationships (OR = 1.495, 95% CI 1.044–2.141) was associated with an increased odds of a female learner being SARU.

### Multivariate analysis: Risk factors associated with a negative sexual health outcome

Adjusting for possible confounding, the multivariate analysis identified factors associated with increased odds of being SARU rather than SARS. We identified factors at the individual, partner/peer and community level. Of the factors included in the multivariate analysis, having more than one prior HIV test (aOR = 2.216, 95% CI 1.396–3.517), thinking that a female partner (aOR = 2.743, 95% CI 1.209–6.225) or both partners (aOR = 2.435, 95% CI 1.487–3.985) were responsible for using contraception, and having a medium level versus low level of condom knowledge (aOR = 1.487, 95% CI 1.085–2.038) were associated with increased odds of being SARU. A decrease in the odds of SARU was associated with being 18 years and older (aOR = 0.258, 95% CI 0.146–0.454), and thinking that it was important not to fall pregnant at school (aOR = 0.636, 95% CI 0.954–0.029).

## Discussion and conclusion

For young women, variables at an individual level[13] appeared to influence whether or not young women had a SARU profile. A key challenge to exploring the influence of partners on a SARU profile outcome was the missing data on key variables relating to sexual partners amongst female learners. As this is an exploratory analysis, we highlight the important variables found during the unadjusted analysis, in particular those factors from the relational context. Overall, the findings suggest that young women may be similarly exposed to broader contextual risk variables (such as quality of schooling, access to services, levels of poverty and social norms around sex in young people), and that it is the individual decisions and context of their sexual relationships which mediate their sexual risk profile outcome [1,3,22,24].

While our findings are consistent with previous research[1,11,22,24,40] suggesting that a greater proportion of older female learners have experienced a negative sexual health outcome, we found some interesting nuances regarding age. Unlike trends seen in antenatal and youth surveys[2,41], or in the previous analysis which include all female learners in this cohort[9,37], when adjusting for confounders, being older decreased the odds of an SARU profile amongst sexually active female learners. While unexpected, this may suggest that when a young woman manages to avoid negative sexual health outcomes when she is younger (under 18), her chances of experiencing a negative outcome diminish as she gets older. This could be for a variety of reasons, including greater sexual autonomy within a relationship[8,42], an increased awareness of how to prevent undesired sexual outcomes, social sanctioning of the use of prevention methods (i.e. contraception, condoms etc.) or sexual relationships with a smaller age-disparity[42,43]. It is also possible that as young women get older and experience a negative SRH outcome they are more likely to drop-out of school, leaving those at lowest risk in school. It stresses the need to ensure safe sex practices amongst younger women with the aim of reducing negative health outcomes as they get older.

Research suggests that partners and peers were important influences on the odds of having a SARU profile. This influence of peers can be both beneficial and negative, by either promoting health services[44–46] or influencing poor adolescent health decision-making[19,47]. We found no peer variables that were associated with increased odds of a SARU profile in the multivariate analysis. While peer pressure to become sexually active reduced the odds of negative health outcomes in young women in the unadjusted analysis it did not remain significant in the multivariate analysis. This may suggest that peers exert an indirect effect on risk by influencing beliefs and social norms around sexual health, but that their influence is limited within the context of an individual’s actual sexual practices. Possibly alluding to the importance of partner or peer influence, believing the responsibility of contraception was that of the female, or both partners was associated with increased odds of a negative SRH profile. This is possibly the result of two relationship types, firstly, one in which the responsibility of protection is placed on the female partner, or one in which a couple who have experienced negative health outcomes make a decision to protect themselves. While further research to investigate these findings is required, these findings highlight the need for better approaches for facilitating the inclusion of the dyad (both the young women and her partner) in sexual health research. At a familial level, unlike previous analysis[9] in all young women in this cohort, we did not find the experience of more than one adult death to be associated with having a SARU profile.

The adjusted analysis highlighted the importance of individual decisions and beliefs about sexual health in affecting a negative SRH profile. Interestingly, we found that a medium level condom knowledge when compared to low condom knowledge, as well as having more than one HIV test in the past, were associated with increased odds of having a SARU profile. Considering exposure to condom education within schools, and that many females had already experienced a negative sexual health outcome which may encourage linkage to care, it is possible that they had some knowledge and access to information about condoms[48]. When compared against low condom knowledge, high condom knowledge was not associated with negative SRH outcomes which may suggest, that information on its own has limited impact on negative SRH outcomes. The experience of multiple HIV tests may be a result of having experienced a negative SRH outcome, or an awareness of risk amongst some sexually active learners. Increased risk perception has been found to be associated with HIV testing uptake amongst adolescents in multiple studies[49]. Discussions with adolescents accessing mobile health services at the CAPRISA clinic (unpublished) have shown that many adolescents will access HIV testing after engaging in a perceived risky sexual encounter, testing more regularly than those who perceive their sexual encounters as low risk. This highlights the importance of HIV testing, not as only an opportunity for increasing awareness of status, but as an opportunity to assess adolescent risk, test for other important SRH outcomes, and for providing a tailored service that is responsive to the lived experience of the adolescent. It is clear that adolescents need SRH services that use contextualised counselling approaches (as opposed to the current and predominant standardised approaches) which assess multiple SRH and social risk outcomes. This can be used to create and understand the risk profile of an adolescent which can be used to respond to their most critical SRH needs, and in-turn reduce their risk of future negative SRH outcomes.

For young women, avoiding pregnancy while at school was associated with reduced odds of SARU profile. Considering the high prevalence of pregnancy amongst young women[9], its link to school drop-out[50], poor health outcomes[51] and its association with increased HIV risk it is an important SRH outcome to reduce in young women. Pregnancy is, arguably, a more tangible fear than HIV for many young women. Whereas an STI (such as HIV) is mostly unobservable by others and, when properly managed, has no impact on future goals, pregnancy is a highly visible sexual health outcome, often impacting on future goals (i.e. attending university, economic autonomy) and may result in negative social outcomes with family and community members[17,18,52]. Therefore, young women who are particularly fearful of getting pregnant may take extra precautions for protecting themselves, thereby indirectly avoiding other negative SRH outcomes. In an age of HIV treatment, with the fear of HIV diminished amongst many young people, using interactions involving other SRH outcomes as opportunities to promote behaviours that may indirectly reduce HIV infection should not be wasted. Viewing adolescents within the paradigm of a risk profile, and promoting SRH services that respond to all important health outcomes facing adolescents, rather than focusing on only HIV offers opportunities to better respond to the SRH needs of adolescents.

Although excluded due to high missing data, the unadjusted analysis raised interesting hypotheses that require further analysis when investigating SARU profiles in other cohorts. Firstly, early sexual experimentation seemed to increase the odds of negative sexual health outcomes. We saw an increase in the odds of a SARU profile amongst those female learners that were under 18 when they first had oral sex, initiating sexual experimentation at an early age. Investigating if adolescents who engage in some form of sexual activity at an early age progress to other forms of sex faster than those who delay any sexual experimentation until they are older needs to be assessed [53]. Sexual partner age has been identified as an important HIV risk variable[3,23]. Our unadjusted analysis suggested that the presence of a recent partner that is four or more years older increases the odds of adolescent females experiencing a negative SRH outcome[22] but whether this is an important risk factor, or a mediator for experiencing a SARU profile, as seen in HIV risk, requires further investigation [3,54].

Due to the cross-sectional nature of the data in this analysis, cautious interpretation of the findings is needed. This study did not collect in-depth and targeted data on all familial and school resilience factors and so the impact these ecological risk variables have on adolescent risk-profiles requires further exploration. Our findings highlight the need to look at these factors across time in order to better explain some of the findings from this exploratory analysis, this is the next step that will be undertaken. Our risk profiles take into account important biological measures that indicate high risk in addition important self-reported SRH risk variables. It is possible that the self-reported SRH variables may overestimate the group of at-risk adolescents due to the limitations of self-report measures. Due to the large amounts of missing data on many of the variables, further research is warranted. While our analysis provides an interesting starting point to develop more nuanced understandings of risk differences amongst female learners, the limitations of the self-collected data means our results require confirmation in future studies, and to see if they change in different cohorts.

Young women are a vulnerable population; their risk extends beyond HIV infection and includes the high risk of several SRH outcomes that negatively impact their sexual health. To reduce the vulnerability of negative SRH outcomes in young women it is clear that we cannot continue with business as usual, conceptualising risk as a profile, not only as the experience of individual SRH outcomes. Our findings highlight that in high-risk setting, where broader ecological variables may be similar, the individual decisions young women make regarding their sexual health and the characteristics of their sexual relationships are important to having a negative SRH profile. Critical to changing how we approach adolescent prevention efforts is to 1) consider the risk profile of adolescents, consider all important negative SRH outcomes, and preventing them from either happening again, or progressing to the experience of multiple SRH outcomes, 2) include partners, extending our prevention efforts to understand the sexual and relational context of young women, and 3) involve parents, schools and communities as key stakeholders in supporting the provision of SRH services, but recognise the autonomy of young women to access SRH services independently. By rethinking adolescent vulnerability, moving beyond HIV to include broader SRH outcomes, we can start targeting at-risk girls, improving the SRH profiles of young women and reducing their risk of negative SRH outcomes.

## Supporting information

S1 Table List of ecological variables assessed as potential risk variables associated with SARU profile. (DOCX)

S2 Table Basic behavioural characteristics of sexually active students in rural KwaZulu-Natal, South Africa. (DOCX)

S3 Table Additional ecological variables analysed using univariate analysis indicating the ecological factors associated with risk profile amongst learners. (DOCX)

S1 File Supporting information female questionnaire baseline. (PDF)
