# Supplementary material for: Exploratory analysis of the ecological variables associated with sexual health profiles in high-risk, sexually-active female learners in rural KwaZulu-Natal
Source: PLoS One. 2018 Apr 5;13(4):e0195107. doi: 10.1371/journal.pone.0195107 (PMC5886415; doi:10.1371/journal.pone.0195107)
Supplement: S1 Table — (DOCX) [file pone.0195107.s001.docx]

**S1 Table: List of Ecological Variables assessed as potential risk variables associated with SARU profile**

| **Individual level** | **Description of variables** |
| --- | --- |
| Age | Median Age, Age category (under 15/16-17/18-19/over 20), Age category (<18, ≥18 years old) |
| Perceived risk | Self-assessment of risk as either low risk or high risk |
| Substance Use | Ever used alcohol (yes/no), cigarettes (yes/no) or other drugs (yes/no) |
| HIV fatigue | Summary variable assessing if female learners are tired of hearing about/learning about HIV |
| Repeated a grade | Ever repeated a grade (Yes/No) |
| Contraception use | Current and ever use contraception (yes/no) |
| Self-efficacy | Summary variable (maximum score 17): Includes questions assessing self-efficacy including questions on efficacy of young people to achieve health, school and future goals. Includes items self-efficacy of protecting yourself against HIV infection and self-efficacy to make sexual decisions in relationships. |
| Fatalism | Summary variable (max score 3): assesses belief that young people in the study community will inevitable get HIV no matter what they do to protect themselves. |
| Important of pregnancy | Importance of not falling pregnant at school (yes/no) |
| Concurrent partners | Have they ever had a more than one partner at the same time (yes/no) |
| Ever HIV test | Have they ever had an HIV test? (yes/no) |
| Number of HIV tests | How many HIV tests have they had? |
| Locus of control | Summary variable assessing who has greatest influence of achievement in schools and goals, either themselves or others. High score suggested external locus of control, while high school suggested internal locus of control. |
| Transactional sex | Have they ever had sex for money, gifts, food airtime? (Yes/No) |
| Age first sex | Age the first had vaginal/anal/oral sex? |
| Attitudes to sexual debut | Age that it is ok to start having vaginal sex (categorised into age groups) |
| Trusted sources of Information on HIV | Trust/don’t trust responses for questions relating to trustworthiness of sources of information including family, peer/partner/health care professionals/traditional leadership/schools/media/government. |
| Correct beliefs about HIV | Summary variable of correct responses to 19 questions about HIV (max score 19). Categorised into low/medium/high categories. |
| Experience of sex | Have they ever experienced vaginal or oral or anal sex (yes/no) |
| Type of sex | Type of sex (oral, anal,vaginal)they have ever had and Type of sex at last sex act. Note that some learners self-reported no to all type of sex and were coded as none |
| Had sex and No. of sex acts in last 30 days | Assessed if they had sex in last 30 days/ assessed how many sex acts in last 30 days. |
| Accuracy of condom knowledge | Summary variable of correct responses to 14 questions about condom use. Categorised into low/medium/high categories. |
| Condom use | Condom use at last sex act (yes/no) |
| Future aspirations | Summary variable of 3 questions assessing if learners think about, plan, and work towards meeting their goals. |
| Number of partners | Number of partners ever (0,1, >1) and number of new partners (0,1, >1) |
| **Partner/Peer** | |
| Gender of friends | Gender of friends? Male/female/both male and female |
| Experiencing Force to have sex | Experience of any form of reported force to have sex. Summary variable: Has anyone used violence/or the threat of violence to force you to have sex? (Yes/No) and What were your reasons for having oral, vaginal or anal sex? Option response: I was forced to. |
| Pressure to have sex | Has participant ever felt pressured by 1) Partner or 2) peer to have sex? (yes/no) |
| Partner status | Does participant know partners status (yes/no) |
| Partner concurrency | Does partner have other partners (yes/no/do not know) |
| Contraception responsibility | Who is responsible for contraception (not important/female/male/both partners/ other) |
| Oldest partner age | Age of oldest partner they had sex with ever |
| Last and current Partner age | Age of last and current partner who they had sex with |
| Partner circumcised | Circumcised partner: Is partner circumcised? (yes/no/don’t know) |
| **Family level** | |
| Head of household | List of who they consider the head of household. |
| Grant Access | How many grants are accesses (0, 1 more than 1, do not know) |
| SES | Summary variables of 9 questions acting as proxy for socio-economic status regarding family’s ability to afford school/uniforms and if they have a cell phone and receive money from their parents as an allowance. |
| Family beliefs on early pregnancy | Family approves/does not approve of them getting pregnant (female) or making someone pregnant (male) |
| No of family deaths | Number of adult deaths in last 12 months |
| Dependants | Number of dependants in the household |
| **School** | |
| Importance of extramural participation | Importance of participation in extramural activities (Not important/important) |
| Connection to school | Summary variable of 3 variables relating to bullying at school/feeling safe at school/and hating school (all yes/no) |
| Structural barriers in school | Summary variable of 6 variables including access to resources/crowded classes/school quality/and bullying. |
| Importance of school attendance | Is it important to go to school each day (yes/no) |
| Poor quality of school | Participants perception of poor school quality (yes/no) |
| Social participation in school | Involved in school activities (no/yes) |
| Feel safe at school | Do they feel safe at school (no/yes) |
| **Community/Service Use** | |
| Gender equality in relationships | Women are able to have an equal say in relationships (yes/no) |
| Traditional healer usage | Do participants use traditional healers? (yes/no) |
| Social participation | Participation in social activity (yes/no), additional variables included ask about participation in church activity and community activity (yes/no) |
| Important issues in community | Important issues in community including crime (yes/no), economic (no/yes) or drug/alcohol problems (no/yes) |
| Medical service used most in community | Learners use of health services including none, clinic in the area, clinic further away, clinic in town, hospital or other/multiple. |
| Comfort using health services | Do they feel comfortable using healthcare services (no/yes) |
