# Supplementary material for: Exploratory analysis of the ecological variables associated with sexual health profiles in high-risk, sexually-active female learners in rural KwaZulu-Natal
Source: PLoS One. 2018 Apr 5;13(4):e0195107. doi: 10.1371/journal.pone.0195107 (PMC5886415; doi:10.1371/journal.pone.0195107)
Supplement: S2 Table — (DOCX) [file pone.0195107.s002.docx]

**S2 Table: Basic behavioural characteristics of sexually active students in rural KwaZulu-Natal, South Africa**

| **Basic Demographics Female** | | | | | | | |
| --- | --- | --- | --- | --- | --- | --- | --- |
| **Variable** | | **Female Overall** | **Female SARU** | | **Female SARS** | | **p-value** |
|  | | **unadjusted % (n/N)** | **unadjusted % (n/N)** | **adjusted %** | **unadjusted % (n/N)** | **adjusted %** |  |
| **Sexual Behaviour** |  | N=587 | 66.1(N=388) |  | 33.9(N=199) |  |  |
|  | **Experience of peno-vaginal sex** | 52.3(307) | 53.1(206) | 49.2 | 50.7(101) | 52.9 | 0.602 |
|  |  | N=570 | 65.8(N=375) |  | 34.2(N=195) |  |  |
|  | **Experience of anal sex** | 3.2(18) | 2.93(11) | 2.8 | 3.59(7) | 5.5 | 0.801 |
|  |  | N=575 | 65.9(N=379) |  | 34(N=196) |  |  |
|  | **Experience of oral sex** | 15.0(86) | 11.6(44) | 10.7 | 21.43(42) | 24.4 | **0.005** |
|  | **Age of sexual debut** | 16yo(15-17) | 16(15-17) | n/a | 16(15-17) | n/a | 0.233 |
|  | **Median age at 1^st^ experience of oral sex** | 15(14-16) | 15(14-17) | n/a | 15(14-16) | n/a | 0.286 |
|  | **Median age at 1^st^ Experience of anal sex** | 16(14-18) | 16(14-18) | n/a | 16(14-17) | n/a | 0.580 |
|  | **Threat of violence for sex** | 17.2 (102) | 17.1(67) | 16.2 | 17.4(35) | 17.1 | 0.909 |
| **HIV status** |  |  |  |  | n/a | n/a | n/a |
|  | **Positive** | 14.8 (88/596) | 22.3 (88/395) | 21.9 | n/a | n/a | n/a |
| **STI** |  |  |  |  | n/a | n/a | n/a |
|  | **Yes** | 18.6 (110/593) | 27.9 (110/394) | 28.7 | n/a | n/a | n/a |
| **HSV_2** |  |  |  |  | n/a | n/a | n/a |
|  | **Positive** | 24.8 (148/596) | 37.5 (148/395) | 38.6 | n/a | n/a | n/a |
| **Pregnancy** |  |  |  |  | n/a | n/a | n/a |
|  | **Positive** | 8.3 (49/592) | 12.5 (49/391) | 12.5 | n/a | n/a | n/a |
|  | **Ever pregnant** |  | 51.9 (147/283) |  | n/a | n/a | n/a |
