# Supplementary material for: Exploratory analysis of the ecological variables associated with sexual health profiles in high-risk, sexually-active female learners in rural KwaZulu-Natal
Source: PLoS One. 2018 Apr 5;13(4):e0195107. doi: 10.1371/journal.pone.0195107 (PMC5886415; doi:10.1371/journal.pone.0195107)
Supplement: S3 Table — (DOCX) [file pone.0195107.s003.docx]

| **S3 Table: Additional variables analysed using univariate analysis indicating the Ecological Factors associated with Risk profile amongst learners in rural KwaZulu-Natal, South Africa** | | | | | | | |
| --- | --- | --- | --- | --- | --- | --- | --- |
|  | |  | **Female Univariate Analysis** | | | | |
|  | | **% (n/N)** | **Odd Ratio** | **CI (95%)** | | | **Sig.** |
| **Characteristic** | |  |  | **Lower** | **Upper** | |  |
| **Individual Level Factors** | | | | | | | |
| **Perceived Risk** | **No Risk** | 27.2% (143/525) | 1.00 (Ref) |  |  | |  |
|  | **At Risk** | 72.8% (382 /525) | 0.970 | 0.699 | 1.344 | | 0.853 |
| **Used Cigarettes** | **No** | 95.6% (560/586) | 1.00 (Ref) |  |  | |  |
|  | **Yes** | 4.4% (26/586) | 0.692 | 0.338 | 1.420 | | 0.316 |
| **Other Drug Use** | **No** | 82.7% (487/589) | 1.00 (Ref) |  |  | |  |
|  | **Yes** | 17.3% (102/589) | 0.985 | 0.666 | 1.456 | | 0.940 |
| **HIV Fatigue** | **Low** | 38.6% (229/593) | 1.00 (Ref) |  |  | |  |
|  | **Medium** | 23.3% (138/593) | 0.71 | 0.414 | 1.218 | | 0.214 |
|  | **High** | 37.9% (226/593) | 0.977 | 0.674 | 1.416 | | 0.900 |
| **Fatalistic Beliefs about HIV infection** | **None** | 11.4% (67/588) | 1.00 (Ref) |  |  | |  |
|  | **Medium** | 69.7% (410/588) | 0.837 | 0.456 | 1.538 | | 0.566 |
|  | **High** | 18.9% (111/588) | 1.061 | 0.473 | 2.382 | | 0.886 |
| **Currently using contraception** | **No** | **57% (158/277)** | **1.00 (Ref)** |  | |  |  |
|  | **Yes** | **43% (119/277)** | **0.561** | **0.287** | | **1.096** | **0.091** |
| **HIV Test ever** | **No** | **45.1% (263/583)** | **1.00 (Ref)** |  | |  |  |
|  | **Yes** | **54.9% (320/583)** | **2.256** | **1.712** | | **2.971** | **<0.000** |
| **Age 1st oral sex** | **<18** | **76.9% (83/108)** | **1.00 (Ref)** |  | |  |  |
|  | **>=18** | **23.1% (25/108)** | **3.072** | **1.319** | | **7.155** | **0.009** |
| **Age 1st Vaginal sex** | **<18** | 58.8% (173/294) | 1.00 (Ref) |  | |  |  |
|  | **>=18** | 41.2% (121/294) | 1.274 | 0.707 | | 2.294 | 0.42 |
| **Age 1st Anal sex** | **<18** | 72.4% (21/29) | 1.00 (Ref) |  | |  |  |
|  | **>=18** | 27.6% (8/29) | 6.625 | 0.525 | | 83.566 | 0.144 |
| **Age 1st oral sex*** | **<18** | **76.9% (83/108)** | **1.00 (Ref)** |  | |  |  |
|  | **>=18** | **23.1% (25/108)** | **3.072** | **1.319** | | **7.155** | **0.009** |
| **Sex Type Ever*^#^** | **Self-reported as None** | 18.7% (78/418) | 1.00 (Ref) |  | |  |  |
|  | **Oral only** | 12% (50/418) | 0.776 | 0.435 | | 1.382 | 0.389 |
|  | **Vaginal only** | **58.6% (245/418)** | **4.068** | **1.95** | | **8.488** | **<0.001** |
|  | **Other (anal, or combination of sex types)** | **10.8% (45/418)** | **4.392** | **2.004** | | **9.623** | **<0.001** |
| **Sex Type last sex act*^#^** | **Self-reported None** | 16.8% (70/417) | 1.00 (Ref) |  | |  |  |
|  | **Oral only** | 11.5% (48/417) | 0.699 | 0.407 | | 1.2 | 0.194 |
|  | **Vaginal only** | **64.7% (270/417)** | **3.609** | **1.89** | | **6.891** | **<0.001** |
|  | **Other(anal, or combination of sex types)** | **7% (29/417)** | **2.044** | **1.086** | | **3.847** | **0.027** |
| **Sex in last 30 days** | **No** | 12.8% (33/258) | 1.00 (Ref) |  | |  |  |
|  | **Yes** | 87.2% (225/258) | 1.259 | 0.503 | | 3.152 | 0.622 |
| **No sex acts in last 30 days** | **none** | 12.8% (33/258) | 1.00 (Ref) |  | |  |  |
|  | **1-2 sex acts** | 26.7% (69/258) | 0.848 | 0.29 | | 2.486 | 0.764 |
|  | **2-4 sex acts** | 14.3% (37/258) | 1.205 | 0.449 | | 3.237 | 0.711 |
|  | **5 or more** | 46.1% (119/258) | 1.626 | 0.644 | | 4.105 | 0.304 |
| **Condom use at last sex act** | **No** | 44.8% (151/337) | 1.00 (Ref) |  | |  |  |
|  | **Yes** | 55.2% (186/337) | 1.402 | 0.636 | | 3.089 | 0.402 |
| **Number of Partners*** | **0** | **14.5% (57/393)** | **1.00 (Ref)** |  | |  |  |
|  | **1** | **51.1% (201/393)** | **1.681** | **1.064** | | **2.658** | **0.026** |
|  | **>1** | **34.4% (135/393)** | **2.319** | **1.43** | | **3.76** | **0.001** |
| **Number of new partner in the last 12 months*** | **0** | **29.9% (118/394)** | **1.00 (Ref)** |  | |  |  |
|  | **1** | **48.7% (192/394)** | **1.805** | **1.063** | | **3.066** | **0.029** |
|  | **>1** | 21.3% (84/394) | 1.672 | 0.865 | | 3.234 | 0.127 |
| **Ever used contraception** | **No** | 63.9% (312/488) | 1.00 (Ref) |  | |  |  |
|  | **Yes** | 36.1% (176/488) | 1.419 | 0.872 | | 2.307 | 0.159 |
| **Concurrent Partners** | **No** | 66% (351/532) | 1.00 (Ref) |  |  | |  |
|  | **Yes** | 34% (181/532) | 0.999 | 0.684 | 1.461 | | 0.998 |
| **Locus of control** | **External** | 17.1%(102/569) | 1.00 (Ref) |  |  | |  |
|  | **Internal** | 82.9% (494/569) | 0.839 | 0.543 | 1.295 | | 0.428 |
| **Transactional sex** | **No** | 87.4% (458/524) | **1.00 (Ref)** |  |  | |  |
|  | **Yes** | 12.6% (66/524) | **1.509** | **0.965** | **2.36** | | **0.071** |
| **Future Aspirations** | **Low future aspirations** | 22.5% (134/595) | 1.00 (Ref) |  |  | |  |
|  | **High Aspirations** | 77.5% (461/595) | 1.164 | 0.802 | 1.689 | | 0.424 |
| **Partner/Peer Level factors** | | | | | | | |
| **Partner Age** | **younger** | 18.2% (65/357) | 1.00 (Ref) |  | |  |  |
|  | **same age** | 14.6% (52/357) | 0.764 | 0.392 | | 1.489 | 0.429 |
|  | **under 4 years** | **43.4% (155/357)** | **0.58** | **0.344** | | **0.976** | **0.04** |
|  | **4-7 years older** | 19.9% (71/357) | 0.582 | 0.255 | | 1.326 | 0.198 |
|  | **8 years older** | 3.9% (14/357) | 0.857 | 0.318 | | 2.312 | 0.761 |
| **Age of last partner** | **younger** | 5.4% (20/371) | 1.00 (Ref) |  | |  |  |
|  | **same age** | 26.1% (97/371) | 1.102 | 0.497 | | 2.442 | 0.811 |
|  | **under 4 years** | 36.7% (136/371) | 1.741 | 0.689 | | 4.396 | 0.241 |
|  | **4-7 years older** | **24.5% (91/371)** | **3.046** | **1.534** | | **6.048** | **0.001** |
|  | **8 years older** | **7.3% (27/371)** | **5.459** | **1.788** | | **16.667** | **0.003** |
| **Oldest Partner Age** | **younger** | 27.4% (110/402) | 1.00 (Ref) |  | |  |  |
|  | **same age** | 12.9% (52/402) | 0.782 | 1.067 | | 0.676 | 1.684 |
|  | **under 4 years** | 38.6% (52/402) | 0.39 | 0.81 | | 0.5 | 1.311 |
|  | **4-7 years older** | 17.7% (155/402) | 0.478 | 0.812 | | 0.458 | 1.442 |
|  | **8 years older** | 3.5% (14/402) | 0.692 | 1.206 | | 0.477 | 3.051 |
| **Partner circumcised** | **None of them** | 49% (196/400) | 1.00 (Ref) |  | |  |  |
|  | **I do not know what circumcision is** | 18.8% (75/400) | 0.915 | 0.509 | | 1.645 | 0.768 |
|  | **Do not know how many have been circumcised** | 17% (68/400) | 0.678 | 0.32 | | 1.435 | 0.309 |
|  | **Some of them** | 7.3% (29/400) | 1.177 | 0.516 | | 2.683 | 0.699 |
|  | **All of them** | 8% (32/400) | 1.224 | 0.581 | | 2.578 | 0.595 |
| **Know Partner status** | **No** | **32.9% (77/234)** | **1.00 (Ref)** |  | |  |  |
|  | **Yes** | **67.1% (157/234)** | **2.134** | **1.233** | | **3.694** | **0.007** |
| **Partner has other partners** | **No** | 23.7% (101/426) | 1.00 (Ref) |  | |  |  |
|  | **Yes** | 11% (47/426) | 1.458 | 0.718 | | 2.959 | 0.297 |
|  | **Don’t know** | 65.3% (278/426) | 0.812 | 0.506 | | 1.305 | 0.39 |
| **Pressure to have sex (partner)** | **No** | 73.1% (381/521) | 1.00 (Ref) |  |  | |  |
|  | **Yes** | 26.9% (140/521) | 0.979 | 0.674 | 1.422 | | 0.911 |
| **Experiencing force to have sex** | **No** | 82.8% (492/594) | 1.00 (Ref) |  |  | |  |
|  | **Yes** | 17.2% (102/594) | 0.99 | 0.664 | 1.478 | | 0.962 |
| **Friends** | **Boys and girls** | 19.7% (111/563) | 1.00 (Ref) |  |  | |  |
|  | **Mostly Girls** | 75.7% (426/563) | 1.198 | 0.741 | 1.938 | | 0.462 |
|  | **Mostly Boys** | 4.6% (26/563) | 0.808 | 0.314 | 2.08 | | 0.658 |
| **Family Level Factors** | | | | | | | |
| **Head of Household** | **Both parents** | 15.3% (91/594) | 1.00 (Ref) |  |  | |  |
|  | **Birth mother** | 35% (208/594) | 0.817 | 0.394 | 1.695 | | 0.587 |
|  | **Birth father** | 13.3% (79/594) | 0.918 | 0.32 | 2.638 | | 0.874 |
|  | **CHH** | 1.3% (8/594) | 0.735 | 0.174 | 3.097 | | 0.675 |
|  | **Grandparent** | 18% (107/594) | 0.996 | 0.384 | 2.579 | | 0.993 |
|  | **Sibling older than 18** | 2% (12/594) | 0.61 | 0.153 | 2.436 | | 0.485 |
|  | **Other** | 15% (89/594) | 0.749 | 0.302 | 1.856 | | 0.532 |
| **Number of adult deaths in Household** | **0** | 45.9% (271/591) | 1.00 (Ref) |  |  | |  |
|  | **1** | 20% (118/591) | 1.116 | 0.766 | 1.626 | | 0.567 |
|  | **>1** | 34.2% (202/591) | 1.033 | 0.686 | 1.555 | | 0.878 |
| **Number of dependants in Household** | **<=3** | 49.9% (296/593) | 1.00 (Ref) |  |  | |  |
|  | **>3** | 50.1% (297/593) | 0.886 | 0.657 | 1.194 | | 0.427 |
| **Grant Access** | **None** | 12.9% (77/595) | 1.00 (Ref) |  |  | |  |
|  | **One** | 45.9% (273/595) | 1.28 | 0.735 | 2.227 | | 0.383 |
|  | **more than one** | 32.9% (196/595) | 1.267 | 0.771 | 2.083 | | 0.35 |
|  | **do not know** | 8.2% (49/595) | 0.894 | 0.537 | 1.489 | | 0.667 |
| **SES** | **Low** | 17.5% (104/595) | 1.00 (Ref) |  |  | |  |
|  | **Medium** | 39.3% (234/595) | 0.982 | 0.549 | 1.757 | | 0.951 |
|  | **High** | 43.2% (257/595) | 1.19 | 0.759 | 1.865 | | 0.448 |
| **Family attitudes to early pregnancy** | **Not approved of** | 77.8% (458/589) | 1.00 (Ref) |  |  | |  |
|  | **Approved of** | 22.2% (131/589) | 0.815 | 0.577 | 1.151 | | 0.246 |
| **School Level Factors** | | | | | | | |
| **Connection to school** | **Poor connection to school** | 11.4% (68/595) | 1.00 (Ref) |  |  | |  |
|  | **Good connection to school** | 88.6% (527/595) | 0.987 | 0.679 | 1.433 | | 0.944 |
| **Structural Barriers in school** | **Minimal barriers** | 18.2% (108/595) | 1.00 (Ref) |  |  | |  |
|  | **High number of barriers** | 81.8% (487/595) | 1.066 | 0.708 | 1.604 | | 0.76 |
| **Important to go to school each day** | **No** | 2.4% (14/590) | 1.00 (Ref) |  |  | |  |
|  | **Yes** | 97.6% (576/590) | 1.091 | 0.443 | 2.686 | | 0.849 |
| **I do not feel safe at school** | **No** | 92.8% (552/595) | 1.00 (Ref) |  |  | |  |
|  | **Yes** | 7.2% (43/595) | 0.8679 | 0.530 | 1.421 | | 0.573 |
| **Social participation in School** | **No** | 1% (6/595) | 1.00 (Ref) |  |  | |  |
|  | **Yes** | 99% (589/595) | 0.985 | 0.176 | 5.519 | | 0.987 |
| **Importance of extramural activity** | **Not important** | 17% (99/583) | 1.00 (Ref) |  |  | |  |
|  | **Important** | 83% (484/583) | 0.874 | 0.515 | 1.485 | | 0.619 |
| **Community Level factors** | | | | | | | |
| **Crime important community issue** | **No** | 66% (388/588) | 1.00 (Ref) |  |  | |  |
|  | **Yes** | 34% (200/588) | 0.892 | 0.635 | 1.253 | | 0.51 |
| **Economic important community issue** | **No** | 13.6% (80/590) | 1.00 (Ref) |  |  | |  |
|  | **Yes** | 86.4% (510/590) | 1.145 | 0.756 | 1.735 | | 0.523 |
| **Drug/alcohol Abuse important community issue** | **No** | 63.3% (373/589) | 1.00 (Ref) |  |  | |  |
|  | **Yes** | 36.7% (216/589) | 0.849 | 0.609 | 1.185 | | 0.337 |
| **Medical Service used most often** | **none** | 4.1% (24/585) | 1.00 (Ref) |  |  | |  |
|  | **clinic in your area** | 69.2% (405/585) | 1.494 | 0.666 | 3.351 | | 0.33 |
|  | **clinic further away in your area** | 4.1% (24/585) | 2.187 | 0.735 | 6.51 | | 0.16 |
|  | **clinic in town** | 7.4% (43/585) | 1.191 | 0.683 | 2.076 | | 0.538 |
|  | **Hospital** | 4.1% (24/585) | **3.518** | **1.102** | **11.23** | | **0.034** |
|  | **other or multiple** | 11.1% (65/585) | 0.81 | 0.407 | 1.614 | | 0.549 |
| **Comfort using services** | **No** | 21.7% (122/561) | **1.00 (Ref)** |  |  | |  |
|  | **Yes** | 78.3% (439/561) | **1.609** | **1.018** | **2.542** | | **0.042** |
| **Traditional Healer** | **No** | 64% (373/583) | 1.00 (Ref) |  |  | |  |
|  | **Yes** | 36% (210/583) | 1.269 | 0.862 | 1.87 | | 0.228 |
| **Community Activity participation** | **No** | 89.5 (535/595) | 1.00 (Ref) |  |  | |  |
|  | **Yes** | 10.1 (60/595) | 0.786 | 0.531 | 1.162 | | 0.227 |
| **Church Activity Participation** | **No** | 75.4 (448/594) | 1.00 (Ref) |  |  | |  |
|  | **Yes** | 24.6 (146/594) | 0.878 | 0.567 | 1.359 | | 0.559 |

***excluded due to high missing data**

**^#^ Type of sex refers to oral, vaginal, or other (anal, or combination of different sex types), some learners reported no to all types of sex and were coded as self-reported as none**
