# Supplementary material for: Exploratory analysis of the ecological variables associated with sexual health profiles in high-risk, sexually-active female learners in rural KwaZulu-Natal
Source: PLoS One. 2018 Apr 5;13(4):e0195107. doi: 10.1371/journal.pone.0195107 (PMC5886415; doi:10.1371/journal.pone.0195107)
Supplement: S1 File — (PDF) [file pone.0195107.s004.pdf]

CAPRISA 007 Baseline Assessments for Female Learners

For Official Use Only

**Participant ID**	0 0 7 - _ _ - _ _ _ _ (Study / Site / Participant)	**Gender**	Male ☐ Female ☒
**School Name**	______________________	**Visit Code**	1 0 0 0
**Staff Initials**	_ _ _	**Date**	_ _ (*dd*) _ _ _ (*MMM*) _ _ (*yy*)

Thank you for agreeing to be part of the CAPRISA 007 study. As part of the study, we would like you to fill in this questionnaire. There are no wrong or right answers to the questions in this form, and it is not a test. The questionnaire is divided into different sections. The sections look at different things that affect you as a young person, these things include: information about how you live, how you feel about school, how you think about things in the future, what after-school activities you do, issues around your health and other young people's health, as well as how you feel about HIV/AIDS.

We would like you to answer all the questions in the questionnaire, as it helps us understand how and what issues are affecting young people in your community. DO NOT write your name on this questionnaire.
Your answers will be kept private. No one will know what you write. Please feel free to answer the questions based on what you really do feel and experience. Please know that all information that you write on these questionnaires will be stored safely and not shared with others, and that any reports that are made as part of the study will not identify you.

Your answers might help us to make the quality of education, social services and health care for people in your age group and community better and perhaps in other similar communities as well. It will also help us know more about how young people understand HIV risk in rural KwaZulu-Natal.

<u>NOTE:</u>

PLEASE USE A DARK PEN and answer by writing an X in the box of your choice. Some questions might need you to write down a number (the question will say please write number) or answer, so please read every question carefully before answering it. Please note that you may skip particular questions if you don't want to answer them but we hope you can answer as many questions as possible.

NB : This page not to be faxed.

CAPRISA 007 Plate 001

Visit Code 1 0 0 0

F

Participant ID 0 0 7 - __ __ - __ __ __ __
Study Site Participant

Introductory Section
Page 1 of 2

Visit Date __ __ / __ __ __ / __ __
dd *MMM* *yy*

X

In this first group of questions, we will learn to answer the types of questions you will have to answer today, to make sure you are comfortable. We strongly encourage you ask questions if you are not sure how to answer. However, please put your correct information in the answers to these questions as we go through them as these are still part of the questionnaire.

a. Please give us your age or the date you were born ? **Please write in a number**

Age __ __ *years* **OR** Date of Birth __ __ / __ __ / __ __
dd *MM* *yy*

e.g. 12 March 1995 would be 1 2 0 3 9 5

b. What language do you speak the most at home ?

mark only one

Zulu ☐

English ☐

Other ☐

Specify ________________________

c. What grade are you in now ?

Grade 9 ☐

Grade 10 ☐

d. How many years have you been in school ? **Please write in a number**

__ __ Years

Version 1 . 0

Date __ __ / __ __ __ / __ __
dd *MMM* *yy*

Staff Initials __ __ __

CAPRISA 007 Plate 002

Visit Code 1 0 0 0

A

Participant ID

0 0 7 - ☐☐ - ☐☐☐☐

Study Site Participant

Introductory Section
Page 2 of 2

e. Have you ever repeated a grade in school ?

Yes ☐ No ☐ → **If no, please skip to question g**

f. If yes, what was the reason for repeating a grade ?

Mark all that apply

Did not get good enough marks	☐	Did not feel like attending school anymore	☐
Had to look after a sick relative	☐	Family could not afford to send me to school	☐
I was/made someone pregnant	☐	Other<br>Specify _ _ _ _ _ _ _ _ _ _ _ _ _	☐

g. How many people usually live in your household (including yourself) ? **Please write a number**

**18 or Older**	
Men ☐☐	Women ☐☐
**Younger than 18**	
Boys ☐☐	Girls ☐☐

Version 1 . 0

Date ☐☐ ☐☐☐ ☐☐
dd MMM yy

Staff Initials ☐☐☐

CAPRISA 007 Plate 003

Visit Code 1 0 0 0

A

Participant ID

0 0 7 - _ _ - _ _ _ _
Study Site Participant

Future Goals

First we would like to ask you questions about your thoughts about the future

1.1 What is the most important goal you want to reach in the next 5 years ?

_ _

_ _

1.2 How often do you think about this goal ? **Mark only one**

I spend a lot of time thinking about reaching my goal	☐
I spend some of my time thinking about reaching my goal	☐
I don’t spend much time thinking about reaching my goal	☐

1.3 Do you know what you need to do to meet this goal ? **Mark only one**

Yes, I know exactly what I need to do to reach my goal	☐
I have an idea of what I need to do to reach my goal	☐
No, at this time, I do not know how I will reach my goal	☐

1.4 If I work hard towards my goal, I expect: **Mark only one**

My chances of reaching my goal to be very good	☐
I might reach my goal, but things in life could happen that would stand in my way	☐
My chances of reaching my goal are still not very good	☐

1.5 Of the following options, which one will have the most influence on you reaching your goal ? **Mark only one**

Parents	☐	Teachers	☐
Myself	☐	Brothers / Sisters	☐
Luck / Chance	☐	My community	☐
Other: Specify _ _ _ _ _ _ _ _ _ _ _ _	☐		

Version 1 . 0

Date _ _ / _ _ _ / _ _
dd MMM yy

Staff Initials _ _ _

Participant ID

0 0 7 - _ _ - _ _ _ _

Study Site Participant

Future Goals

1.6 Please read each of the statements below about young women and young men in your community and say if you agree or disagree with them.

When I think about young women in my community, I think it is easy for them to :

Statement	Agree	Disagree
Finish school	☐	☐
Go to university if they want to	☐	☐
Stay HIV negative	☐	☐
Wait until they are ready to have children	☐	☐
Get a job in the community	☐	☐
Eventually earn more than their parents	☐	☐
Control what happens in their life	☐	☐
Have equal say in their relationships with their boyfriend / husband	☐	☐
Get a job that they want	☐	☐
Start their own business	☐	☐
Support themselves without help from their boyfriend / husband	☐	☐
Keep themselves healthy and strong	☐	☐
Give up and not make plans for the future	☐	☐

Version 1 . 0

Date _ _ / _ _ _ / _ _
dd MMM yy

Staff Initials _ _ _

CAPRISA 007 Plate 005

Visit Code 1 0 0 0

A

Participant ID

0 0 7 - _ _ - _ _ _ _
Study Site Participant

Future Goals

1.6 Continued......**When I think about young men in my community, I think it is easy for them to :**

Statement	Agree	Disagree
Finish school	☐	☐
Go to university if they want to	☐	☐
Stay HIV negative	☐	☐
Wait until they are ready to have children	☐	☐
Get a job in the community	☐	☐
Eventually earn more than their parents	☐	☐
Control what happens in their life	☐	☐
Have allow their girlfriend / wives to have equal say in their relationship	☐	☐
Get a job that they want	☐	☐
Start their own business	☐	☐
Earn enough money to support a family	☐	☐
Keep themselves healthy and strong	☐	☐
Give up and not make plans for the future	☐	☐

CAPRISA 007 Plate 006

Visit Code 1 0 0 0

A

Participant ID

0 0 7 - _ _ - _ _ _ _

Study Site Participant

Background Information
Page 1 of 4

Now, we are going to ask you some basic questions about yourself, your family and how you save or spend your money. This section will help us understand more about you and how you live.

2.1 Who would you consider the head of your household ? **Mark only one**

Birth mother	☐	Aunt	☐
Sister / brother 18 years or older	☐	Uncle	☐
Birth father	☐	Grandmother	☐
Another person under the age of 18	☐	Grandfather	☐
Both parents	☐	Myself	☐
Other: Specify _ _ _ _ _ _ _ _ _ _ _ _	☐		

2.2 In your household, how many people have been sick (not in good health including sickness' like flu) in the past 12 months (including yourself) ?

Write in a number

**18 or Older**	
Men ☐☐	Women ☐☐
**Younger than 18**	
Boys ☐☐	Girls ☐☐

2.3 In your household, how many people have died in the past 12 months ?

Write in a number

**18 or Older**	
Men ☐☐	Women ☐☐
**Younger than 18**	
Boys ☐☐	Girls ☐☐

Version 1 . 0

Date ☐☐ ☐☐☐ ☐☐ (dd MMM yy)

Staff Initials ☐☐☐

CAPRISA 007 Plate 007

Visit Code 1 0 0 0

A

Participant ID

0 0 7 - ☐☐ - ☐☐☐☐

Study Site Participant

Background Information
Page 2 of 4

2.4. If there were any deaths in your household in the past 12 months, do you know why they died (e.g. violence, disease, HIV, accident) ?

Yes ☐

No ☐ → **If no, please skip to question 2.6**

No one in my household died ☐ → **please skip to question 2.6**

2.5 If yes what did they die of ?

Sex		What they died of
**Male**	**Female**	**18 or Older**
☐	☐	
☐	☐	
☐	☐	
☐	☐	
☐	☐	
☐	☐	
		**Younger than 18**
☐	☐	
☐	☐	
☐	☐	
☐	☐	
☐	☐	
☐	☐	
☐	☐	

Participant ID

0 0 7 - ☐☐ - ☐☐☐☐

Study Site Participant

Background Information
Page 3 of 4

2.6 How many people from your household are currently in school (including yourself) ?

**Older than 18**	**Grade R- 7**	**Grade 8 - 12**
Men	☐☐	☐☐
Women	☐☐	☐☐
**Younger than 18**		
Boys	☐☐	☐☐
Girls	☐☐	☐☐

2.7 Does anyone in your household (including yourself) access any of the following ?

	**Yes**	**No**	**Do not know**
Child Care / Support Grant	☐	☐	☐
Disability Grant	☐	☐	☐
Old Age Grant (Pension)	☐	☐	☐
Any other type of social grant	☐	☐	☐

2.8 Do you have your own cellphone ?

Yes ☐

No ☐

2.9 Do you work (to earn money) after school / or weekends / or during your holidays ?

Yes ☐

No ☐

2.10 Do you get pocket money ?

Yes ☐

No ☐

CAPRISA 007 Plate 009

Visit Code 1 0 0 0

A

Participant ID

0 0 7 - _ _ - _ _ _ _

Study Site Participant

Background Information
Page 4 of 4

2.11 About how much of your own money do you spend a month ? **Mark only one**

R0 - R50 ☐ R51 - R100 ☐

R101 - R150 ☐ More than R150 ☐

2.12 Do you ever save any money ?

Yes ☐

No ☐ → **If no, please go to question 2.14**

2.13 How do you save money ? **Mark all that apply to you**

Bank account ☐ Save my cash at home ☐

Post office ☐ Other ☐

Specify ____________________

2.14 If you had money that you could save, how would you save this money ? **Mark all that apply to you**

Bank account ☐ Save my cash at home ☐

Post office ☐ Other ☐

Specify ____________________

2.15 Which of these would you save money for ?

Mark all that apply

For airtime	☐	To go out with friends	☐
To go to movies	☐	To buy gifts	☐
To spend money on my partner	☐	To help buy things for the household	☐
To buy clothes	☐	Because it is important to have saved money for emergencies	☐
For University/Technikon/college	☐	Other: Specify: _ _ _ _ _ _ _ _ _ _ _ _ _	☐

Version 1.0

Date _ _ / _ _ _ / _ _
dd MMM yy

Staff Initials _ _ _

CAPRISA 007 Plate 010

Visit Code 1 0 0 0

A

Participant ID

0 0 7 - _ _ - _ _ _ _
Study Site Participant

School Performance and Attendance

In this section, we would like to ask you some questions about your schooling

3.1 Do you think it is important to go to school every day ?

Yes ☐ No ☐

3.2 Please read the statements below about the importance of school for you and tell us if you agree or disagree with each of the following statements:

Statement	Agree	Disagree
I want to go to university therefore I need to do well in school	☐	☐
I spend time after school everyday to try and improve my marks	☐	☐
I am trying to pay more attention in my class	☐	☐
I ask someone like a teacher / friend for help in areas I am having difficulty	☐	☐
I would do better if the teachers were able to control the classes better	☐	☐
I only come to school because my parents tell me to	☐	☐
Coming to school gives me something to do	☐	☐
I would rather be working than coming to school	☐	☐
My marks would be better if my teachers were able to inspire me to study	☐	☐
If I did not have to pay school fees I would perform better at school	☐	☐
Even if I do well in school, it will still be hard for me to find a job	☐	☐
I enjoy the school work I do at school	☐	☐

Visit Code 1 0 0 0

CAPRISA 007 Plate 011

A

Participant ID

0 0 7 - __ __ - __ __ __ __

Study Site Participant

School Performance and Attendance

3.2 Continued

Please read the statements below about the importance of school for you and tell us if you agree or disagree with each of the following statements:

Statement	Agree	Disagree
Getting rewarded for doing well in school would inspire me to do better	☐	☐
If classes were more relevant to life I would enjoy learning more	☐	☐
I do not think that having more time for homework would help me do better at school	☐	☐
I am in control of how well I do in school	☐	☐
Having access to books from a library would make it easier for me to complete school assignments	☐	☐
Having enough textbooks would make it easier for me to learn	☐	☐
I would like support from my family regarding school work	☐	☐
The classrooms are too crowded for me to concentrate	☐	☐
If I didn't have to buy school uniforms it would help me do better at school	☐	☐
Having someone help me understand my school work would help me do better	☐	☐
I find it difficult to learn because there are too many learners in my class	☐	☐
The quality of schooling I am getting is bad	☐	☐

3.3 Please think about how many days you have missed school this year, if you added them up about how many days in total have you been absent ? **Mark only one**

1 - 4 days	☐	5 - 10 days	☐
11 - 20 days	☐	21 - 30 days	☐
More than 30 days	☐	None	☐

Version 1 . 0

Date __ __ / __ __ __ / __ __ (dd MMM yy)

Staff Initials __ __ __

CAPRISA 007 Plate 012

Visit Code 1 0 0 0

A

Participant ID

0 0 7 - _ _ - _ _ _ _
Study Site Participant

School Performance and Attendance

3.4 What are the most common reasons for you missing school ? **Mark all that apply**

I was looking after a sick family member	☐	I cannot get to school	☐
Just didn't feel like going to school	☐	I was pregnant / made someone pregnant	☐
I don't learn anything at school	☐	I could not afford to buy school uniform	☐
I could not afford to buy school stationery	☐	I could not afford to buy school text books	☐
I don't like my teacher	☐	I could not afford to pay school fees	☐
I did not have transport	☐	I was with my partner	☐
I hate school	☐	I overslept	☐
There was death in the family	☐	I was working to make money	☐
I was sick	☐	I went to collect a social grant	☐
I don't feel safe in my school	☐	I had to take care of my brothers and sisters	☐
I was being bullied in school	☐	My parents don't mind if I miss school	☐
Other : Specify _ _ _ _ _ _ _ _ _ _ _	☐		

3.5 What was your **average mark** last year for ? **Please write in a number**

Subject	Marks (%)	Do not know	Not Applicable
English	_ _ _ %	☐	☐
Zulu	_ _ _ %	☐	☐
Life science	_ _ _ %	☐	☐
Physical science	_ _ _ %	☐	☐
Maths	_ _ _ %	☐	☐
Maths literacy	_ _ _ %	☐	☐

Version 1 . 0

Date _ _ / _ _ _ / _ _
dd MMM yy

Staff Initials _ _ _

Participant ID

0 0 7 - [ ][ ] - [ ][ ][ ][ ]

Study Site Participant

School Performance and Attendance

3.6 Do you think this is the best you can do ?

	Yes	No	Not Applicable
English	☐	☐	
Zulu	☐	☐	
Maths / Maths literacy	☐	☐	
Life science	☐	☐	☐
Physical science	☐	☐	☐

3.7 Please read the following paragraph and answer the questions underneath the paragraph :

As you are no doubt aware the flu can strike rapidly and extensively during winter. It can leave its victims ill for weeks. The best way to fight the virus is to have a fit and healthy body. Daily exercise and a diet including plenty of fruit and vegetables are highly recommended to assist the immune system to fight this invading virus. Some schools have decided to offer learners the opportunity to be immunized against the flu as an additional way to prevent this virus from spreading amongst the learners. The school has arranged for a nurse to administer the immunisations, during the school day. This program is free and available to all learners. Participation is voluntary. Learners taking up the option will be asked to sign a consent form indicating that they do not have any allergies, and that they understand they may experience minor side effects. Medical advice indicates that the immunisation does not produce influenza. However, it may cause some side effects such as fatigue, mild fever and tenderness of the arm.

Please answer the following questions according to the paragraph above :

3.7.1 What time of the year do you most often get flu ?

____

3.7.2 How much do you have to pay for an immunisation ?

____

3.7.3 Can you choose not to have an immunisation ?

____

Official Use

1 ☐

2 ☐

3 ☐

CAPRISA 007 Plate 014

Visit Code 1000

A

Participant ID

0 0 7 - __ __ - __ __ __ __

Study Site Participant

School Performance and Attendance

3.7.4 Which one of the following describes a feature of the schools flu immunisation program ?

Mark only one

Daily exercise classes will be run during winter	☐
Immunisations will be given during school hours	☐
A small bonus will be offered to participants	☐
A doctor will give the injections	☐

3.7.5 This paragraph suggests that if you want to protect yourself against the flu virus, a flu injection is :

Mark only one

More effective than exercise and a healthy diet, but more risky	☐
A good idea, but not a substitute for exercise and a healthy diet	☐
As effective as exercise and a healthy diet, and less troublesome	☐
Not worth considering if you have plenty of exercise and a healthy diet	☐

Version 1.0

Date __ __ / __ __ __ / __ __
dd MMM yy

Staff Initials __ __ __

CAPRISA 007 Plate 015

Visit Code 1 0 0 0

A

Participant ID

0 0 7 - ☐☐ - ☐☐☐☐

Study Site Participant

School Performance and Attendance

3.8 The next questions are designed to be like ones you might get in school, we do not expect that everyone will know the answers, but please try your best to answer them.

A medicine dosage pamphlet gives the following rule for determining a child's dosage in terms of the adult dosage:

***Young's rule**: Divide the child's age by the child's age plus 12.* **(** 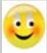 *= the age of the child* **)**

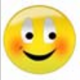

―――――――― = **Number** **This number is the fraction of the adult dose a child needs**

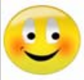 + ***12***

***Example:** dosage for a 4 year old: 4 divided by (4+12) = 1/4 or 0.25 of the adult dosage.*

***Example:** So, if an adult needs 40 drops, how many should a child take?*

Number **X** **ADULT DROPS = number of drops a child needs**

***Example:** 0.25 x 40 adult drops = 10 drops… so a child should take 10 drops*

Answer the questions that follow (which refer to doses of medicine) using this formula.

3.8.1 What fraction of an adult dosage must a 12 year old take?

____

3.8.2 If the adult dosage of a certain medicine is 60 drops, how many drops should an eight year old child be given ?

____

Official Use

1 ☐

2 ☐

Version 1 . 0

Date ☐☐ ☐☐☐ ☐☐ (*dd* *MMM* *yy*)

Staff Initials ☐☐☐

CAPRISA 007 Plate 016

Visit Code 1 0 0 0

A

Participant ID

0 0 7 - _ _ - _ _ _ _

Study Site Participant

School Performance and Attendance

Thabo lives in Johannesburg and is exploring selling ice cream in order to pay for his college fees. He has established the following information:

EXPENSES:
R3 000,00 *monthly payment for the first 12 months to pay for the bicycle and franchise fee.*
R3,50 *per ice cream to the company*
R0,50 *for a serviette and spoon that he supplies with each ice cream*
R0,50 *franchise fee per ice cream to the company*
R25,00 *per day for the block of ice that he uses to keep the container cold*

INCOME:
R10,00 *per ice cream that he sells.*

3.8.3 What are/is Thabo's fixed monthly expenses (expenses that stay the same every month)?

	**Mark the fixed expenses only**
R3 000, 00 monthly payments for the first 12 months to pay for the bicycle and franchise fee	☐
R3, 50 per ice cream to the company	☐
R0, 50 for a serviette and spoon that he supplies with each ice cream	☐
R25, 00 per day for the block of ice that he uses to keep the container cold	☐
R10, 00 Thabo earns per ice cream that he sells	☐
R0, 50 franchise fee per ice cream to the company	☐

3.8.4 What are/is Thabo's variable expenses (expenses that can change every month)?

	**Mark the variable expenses only**
R3 000, 00 monthly payments for the first 12 months to pay for the bicycle and franchise fee	☐
R3, 50 per ice cream to the company	☐
R0, 50 for a serviette and spoon that he supplies with each ice cream	☐
R25, 00 per day for the block of ice that he uses to keep the container cold	☐
R10, 00 Thabo earns per ice cream that he sells	☐
R0, 50 franchise fee per ice cream to the company	☐

Participant ID

0 0 7 - _ _ - _ _ _ _
Study Site Participant

School Performance and Attendance

3.8.5 What are/is Thabo's source(s) of income and say if it is fixed (income that will stay the same every month) or variable (income that can change every month)?

		Type	
	Mark the Income only	Variable	Fixed
R3 000, 00 monthly payments for the first 12 months to pay for the bicycle and franchise fee	☐	☐	☐
R3, 50 per ice cream to the company	☐	☐	☐
R0, 50 for a serviette and spoon that he supplies with each ice cream	☐	☐	☐
R25, 00 per day for the block of ice that he uses to keep the container cold	☐	☐	☐
R10, 00 Thabo earns per ice cream that he sells	☐	☐	☐
R0, 50 franchise fee per ice cream to the company	☐	☐	☐

The company has told Thabo that salesmen typically sell a minimum of 30 ice creams and a maximum of 60 ice creams per day

3.8.6 Will Thabo's variable expenses for a day on which he sells 30 ice creams be R160, 00?

Yes ☐

No ☐

Do not know ☐

3.8.7 Complete the table below about Thabo's monthly income (only write down the values of **a, b and c**)

Monthly Income				
No of days worked in the month	**4**	**8**	**15**	**30**
30 ice creams sold per day	**R 1 200,00**	**a**	**b**	**c**
60 ice creams sold per day	**R 2 400,00**	**R4 800,00**	**R9 000,00**	**R18 000,00**

a ____________________

b ____________________

c ____________________

Official Use
1 ☐
2 ☐
3 ☐

CAPRISA 007 Plate 018

Visit Code 1 0 0 0

A

Participant ID

0 0 7 - [ ][ ] - [ ][ ][ ][ ]
Study Site Participant

HIV Knowledge and Perceived Vulnerability

We would like to ask you a few questions about your community. We will also ask you some questions about HIV, the virus that causes AIDS, and HIV testing.

4.1 Rate how important you think each of the following issues are in **your** community ?

Issue	Not Important	Important	Very Important
HIV/AIDS	☐	☐	☐
Poverty	☐	☐	☐
Crime	☐	☐	☐
Drug abuse	☐	☐	☐
Transport	☐	☐	☐
Unemployment	☐	☐	☐
Teenage pregnancy	☐	☐	☐
Violence	☐	☐	☐
Alcohol	☐	☐	☐

4.2 Would you trust the following people or places for information on HIV/AIDS ?

Source	Trust	Not Trust
My parents	☐	☐
My friends	☐	☐
My boyfriend / girlfriend	☐	☐
My brother / sister	☐	☐
TV / radio	☐	☐
Magazines	☐	☐
Newspapers	☐	☐
Life orientation teacher	☐	☐
Staff from a medical clinic	☐	☐
The principal and teachers at my school	☐	☐
The hospital staff	☐	☐
The *inkosi* and *indunas*	☐	☐

Version 1.0

Date [ ][ ] [ ][ ][ ] [ ][ ]
dd MMM yy

Staff Initials [ ][ ][ ]

CAPRISA 007 Plate 019

Visit Code 1 0 0 0

A

Participant ID

0 0 7 - _ _ - _ _ _ _

Study Site Participant

HIV Knowledge and Perceived Vulnerability

4.2 Continued...Would you trust the following people or places for information on HIV/AIDS ?

Source	Trust	Not Trust
My grandparents	☐	☐
Government	☐	☐
Other: Specify _ _ _ _ _ _ _ _ _ _ _ _ _ _ _ _ _ _ _	☐	☐

4.3 Is there something you can do to avoid getting infected with HIV, the virus that causes AIDS ?

Yes ☐

No ☐ → **If no , please skip to question 4.4**

Don't know ☐ → **If you do not know , please skip to question 4.4**

If yes, please specify what you can do to prevent HIV infection

_ _

_ _

4.4 What percentage of people, **in your community**, do you think are HIV positive in the following age groups:
(for example 68 % means 68 out of 100 people)

Age Group	Percentage
2 - 14 years old	☐☐☐ %
15 - 19 years old	☐☐☐ %
20 - 24 years old	☐☐☐ %
25 - 29 years old	☐☐☐ %
30 - 34 years old	☐☐☐ %
35 - 40 years old	☐☐☐ %
41 - 44 years old	☐☐☐ %
45 - 49 years old	☐☐☐ %
50 and above years old	☐☐☐ %

Participant ID

0 0 7 - [ ][ ] - [ ][ ][ ][ ]

Study Site Participant

HIV Knowledge and Perceived Vulnerability

4.5. Please read the statements about HIV and say if you agree or disagree with them

Statement	Agree	Disgree
Using condoms every time you have sex helps protect against HIV	☐	☐
Being faithful helps to protect you from getting infected with HIV	☐	☐
HIV infected mothers can infect their babies during pregnancy if not treated	☐	☐
Abstaining from sex doesn't protect you from getting HIV/AIDS	☐	☐
Your chances of getting infected with HIV are higher if your boyfriend / girlfriend is older than twenty years old	☐	☐
If the HIV rates are high in your community, you are more at risk of getting infected with HIV if you are having sex	☐	☐
Adolescent girls are at a higher risk of getting infected with HIV compared to adolescent boys	☐	☐
The only way to know your HIV status is to have a HIV test	☐	☐
If a male is circumcised it reduces his chances of getting infected with HIV by about half	☐	☐
Avoiding sex with people who have many partners helps reduce your HIV risk	☐	☐
HIV is a big problem in South Africa	☐	☐
Having unprotected anal sex is about 4 to 7 times more risky than vaginal sex for the receptive partner	☐	☐
If you think your boyfriend / girlfriend is putting you at risk of getting infected with HIV, you have the right to refuse to have sex with them	☐	☐
It is hard for people to protect themselves against HIV	☐	☐

Please continue to next page

CAPRISA 007 Plate 021

Visit Code 1 0 0 0

A

Participant ID

0 0 7 - _ _ - _ _ _ _

Study Site Participant

HIV Knowledge and Perceived Vulnerability

4.5 Continued.... Please read the statements about HIV and say if you agree or disagree with them

Statement	Agree	Disagree
Most people who test HIV positive will still be accepted by their friends and family	☐	☐
Young people would need help to tell their family, if they tested HIV positive	☐	☐
Most young people in this community will eventually get HIV	☐	☐
HIV infected mothers can infect their babies through breastfeeding	☐	☐
If someone is HIV positive there are anti-retroviral drugs available to treat them	☐	☐

4.6. Please read the statements about condoms and say if you agree or disagree with them

Statement	Agree	Disagree
Male condoms protect against sexually transmitted infections including HIV	☐	☐
A woman has the right to ask her boyfriend to use a condom during sex	☐	☐
Male and female condoms are one way to protect against getting HIV	☐	☐
People say that sex feels better without a condom	☐	☐
Not using a condom increases a person’s chance of getting infected with HIV	☐	☐
Male and female condoms are a reliable way of protecting against unwanted pregnancy	☐	☐
People say that a male condom is difficult to use	☐	☐
I have seen a female condom	☐	☐
Male condoms contain HIV	☐	☐

Please continue to next page

Version 1.0

Date ☐ _ _ dd _ _ _ MMM _ _ yy

Staff Initials _ _ _

CAPRISA 007 Plate 022

Visit Code 1 0 0 0

A

Participant ID

0 0 7 - _ _ - _ _ _ _
Study Site Participant

HIV Knowledge and Perceived Vulnerability

4.6 Continued .... Please read the statements about condoms and say if you agree or disagree with them.

Statement	Agree	Disagree
It is embarassing to go buy condoms	☐	☐
It is embarassing to go and get condoms from a clinic	☐	☐
Government condoms are not safe	☐	☐
Most people do not know how to use a female condom	☐	☐
It is not necessary to wear a condom every time you have sex	☐	☐

4.7 Please read the statements about HIV below and say if you agree or disagree with them

Statement	Agree	Disagree
I think that an illness like HIV makes it difficult to think about your future	☐	☐
I am tired of thinking about HIV	☐	☐
I think people are less careful about not getting HIV today, because they are tired of being 'safe'	☐	☐
I often ignore messages about HIV	☐	☐
I have heard enough messages about HIV/AIDS and do not want to hear anymore	☐	☐
Sometimes I do things where I might get HIV because I am tired of being careful	☐	☐
HIV is really not my problem, it is somebody else's	☐	☐
I don't think HIV is a threat to my future because I can get treatment (i.e. ARV's)	☐	☐
I can try and be 'safe', but I will probably get HIV/AIDS anyway	☐	☐

Version 1.0

Date _ _ / _ _ _ / _ _
dd MMM yy

Staff Initials _ _ _

CAPRISA 007 Plate 023

Visit Code 1 0 0 0

A

Participant ID

0 0 7 - -

Study Site Participant

HIV Knowledge and Perceived Vulnerability

HIV Testing
Now we are going to talk about HIV testing. We will not ask you if you have ever tested HIV positive or negative. We will not ask you about any results of any HIV test today, but we will ask you a few questions about HIV testing. Please do not feel you have to share any information that you do not want to.

4.8 When it comes to HIV do you think you are at ?
Mark only one

High risk of getting HIV	☐	Some risk of getting HIV	☐
Low risk of getting HIV	☐	Not at risk of getting HIV	☐

4.9 Do you think you might be HIV positive ? **Mark one only**

Yes ☐ No ☐

Not Sure ☐ I do not want to know ☐

4.10 Before today have you ever had an HIV test ?

Yes ☐ No ☐ → **If no, please skip to question 4.13**

4.11 If yes, when was your last HIV test ?
Mark only one

Within the last 3 months	☐	4-6 months ago	☐
7 - 12 months ago	☐	More than a year ago	☐
Do not remember	☐		

4.12 Before today, how many times have you had an HIV test ? **Please write a number**

☐☐ Times

Version 1 . 0

Date dd MMM yy

Staff Initials

Participant ID

0 0 7 - _ _ - _ _ _ _

Study Site Participant

HIV Knowledge and Perceived Vulnerability

4.13 Please read the following statements about HIV tests and say how much you agree or disagree with them

Mark all that apply

**Statement**	**Agree**	**Disagree**
I think it is important to have a HIV test at least once a year	☐	☐
I don't know where to go to have a HIV test	☐	☐
I am scared to have a HIV test	☐	☐
I don't need to have a HIV test if I haven't had sex	☐	☐
If I am HIV positive I need to make sure I do not infect others	☐	☐
I do not have to share my HIV test results with anyone I don't want to	☐	☐
I'm scared that other people will think I'm HIV+ if they find out I have had a HIV test	☐	☐
The clinic staff know me and my family and therefore I can't go to the clinic to have a HIV test	☐	☐
I think people who have a HIV test and know their status are being responsible	☐	☐

4.14 Where would you prefer to have a HIV test ?

Mark only one

At a clinic closest to my school or home	☐	At a clinic **not** close to my school or home	☐
At a hospital	☐	At school	☐
At a counselling service or mobile testing unit (e.g. Lifeline)	☐	Other<br>Specify _ _ _ _ _ _ _ _ _ _ _ _	☐

Participant ID

0 0 7 - _ _ - _ _ _ _

Study Site Participant

Health Care Utilization

Now we would like to ask you about the medical clinics you use, and why you might use them

5.1. What is the purpose for you visiting a medical clinic? **Mark all that apply**

Sickness	☐	Vaccination (injection)	☐
Contraceptive	☐	Voluntary HIV counseling and testing	☐
Condoms	☐	Taking family members	☐
General check ups	☐	Antenatal services	☐
Health advice	☐	Other<br>Specify _ _ _ _ _ _ _ _ _ _ _ _ _ _	☐

5.2. What medical clinic do you use **most often** when you need medical services? **Mark one only**

Clinic in your area (close to your home)	☐	Clinic in another area further from your home	☐
Clinic in a nearby town (i.e. Howick or Pietermaritzburg)	☐	Hospital	☐
None	☐	→ **If none, please skip to question 5.9**	
Other<br>Specify _ _ _ _ _ _ _ _ _ _ _ _ _	☐		

5.3 Why do you use **this** medical clinic ? **Mark all that apply**

Close to my home	☐	It is cheap	☐
My family uses this health facility	☐	Close to transport links	☐
Friendly staff	☐	Want to go somewhere, where they do not know me	☐
Other<br>Specify _ _ _ _ _ _ _ _ _ _ _ _ _ _	☐		

Participant ID

0	0	7	-			-				

Study Site Participant

Health Care Utilization

5.4. Do you feel comfortable going to this medical clinic when you need to?

Yes ☐ → **If yes, please answer question 5.6**

No ☐ → **If no, please go to question 5.5** and then go to **question 5.7**

5.5. **If no**, why don't you feel comfortable going to this medical clinic? **Mark all that apply**

The staff moan at children who come for condoms / contraceptive	☐	The service is slow	☐
The staff gossip	☐	People will see you at the clinic	☐
I know the staff at the clinic from my community	☐	The staff look down on you	☐
Other<br>Specify _ _ _ _ _ _ _ _ _ _ _ _ _	☐		

Now, please go to question 5.7

5.6 **If yes**, what makes you feel comfortable going to this medical clinic ? **Mark all that apply**

Friendly staff	☐	The staff are good at their jobs	☐
Feel safe	☐	The service is quick	☐
It is private	☐	They have many services	☐
Other<br>Specify _ _ _ _ _ _ _ _ _ _ _ _ _	☐		

CAPRISA 007 Plate 027

Visit Code 1 0 0 0

A

Participant ID

0 0 7 - [ ][ ] - [ ][ ][ ][ ]
Study Site Participant

Health Care Utilization

5.7 When was the last time you went to this medical clinic ?
Mark only only one

Last Month	☐
2 - 3 months ago	☐
4 - 6 months ago	☐
7 - 12 months ago	☐
Over a year ago	☐

5.8. In the last year, how often did you go to this medical clinic? **Please write in a number**

[ ][ ][ ] times

OR

☐ I have not gone to a medical clinic in the last year

5.9. Do you ever visit a traditional healer (i.e. Sangoma, or Inyanga)?

Yes ☐ **If yes, please go to question 5.10**

No ☐ **If no , please go to question 6.1**

5.10. What is the purpose for you visiting the traditional healer? **Mark all that apply**

Treatment for sickness	☐	Taking family members	☐
Contraceptive	☐	Health advice	☐
Condoms	☐	Other<br>Specify _ _ _ _ _ _ _ _ _ _ _ _ _	☐

CAPRISA 007 Plate 028

Visit Code 1 0 0 0

A

Participant ID

0 0 7 - __ __ - __ __ __ __

Study Site Participant

Relationships

I would like to discuss issues related to having a boyfriend or girlfriend. Please read the definitions below and answer these questions as honestly as possible. Your answers will be confidential.

When we talk about having a boyfriend or girlfriend, it can mean someone who you are just 'dating' and not having sex with or someone you are having sex with. A boyfriend/girlfriend can also mean any secret boyfriends/girlfriends that you have.	
**Sex Partner**	Person who you have had oral, vaginal or anal sex with (may be boyfriend/girlfriend, a secret boyfriend/girlfriend or someone who you have sex with that is not a boyfriend/ girlfriend)
When we talk about having sex, we may talk about different types of sex people can have. We may also ask about each type of sex individually. These types of sex are oral, vaginal and anal sex	
**Oral Sex**	Is a type of sex where one person puts their mouth or tongue on their boyfriend / girlfriend's penis or vagina or anus
**Vaginal Sex**	Is a type of sex between a man and a woman, where the man puts his penis into the woman's vagina
**Anal Sex**	The other type of sex is anal sex, which is sex where the man puts his penis through his sexual partner's anus, the opening where #2 comes out. This partner can be a man or a woman. Women and men can have anal sex, and men and men can have anal sex. This is not a sexual position, but a sexual act.

6.1 Have you ever had a boyfriend or girlfriend ?

Yes ☐ No ☐ → **If no, please skip to question 6.5**

6.2 How many different boyfriends / girlfriends have you ever had ? **Please write in a number**

☐☐☐ boyfriends / girlfriends

6.3 Have you ever had more than one boyfriend / girlfriend at the same time ?

Yes ☐ No ☐

6.4 Have you ever felt pressured by your boyfriend / girlfriend to have sex ?

Yes ☐ No ☐

Version 1 . 0

Date dd MMM yy

Staff Initials

CAPRISA 007 Plate 029

Visit Code 1 0 0 0

A

Participant ID

0 0 7 - _ _ - _ _ _ _

Study Site Participant

Relationships

6.5. Have you ever felt pressured by your friends/peers to have sex ?

Yes ☐ No ☐

6.6 Has anyone used violence/or the threat of violence to force you to have sex ?

Yes ☐ No ☐

6.7. At what age do you think it is OK to start having oral, vaginal or anal sex ? **Please write in a number**

Oral Sex ☐☐ Years old

Vaginal Sex ☐☐ Years old

Anal Sex ☐☐ Years old

6.8. Have you ever had oral, vaginal or anal sex?

	Yes	No
Oral Sex	☐	☐
Vaginal Sex	☐	☐
Anal Sex	☐	☐

If you answered YES to any of the three (you have had oral, vaginal, or anal sex) go to SECTION A

If you answered NO to ALL three (you have never had oral, vaginal, or anal sex) go to SECTION B

Version 1 . 0

Date ☐☐ ☐☐☐ ☐☐
dd *MMM* *yy*

Staff Initials ☐☐☐

Visit Code 1 0 0 0

CAPRISA 007 Plate 030

A

Participant ID

0 0 7 - _ _ - _ _ _ _

Study Site Participant

Relationships

SECTION A

6.9. If yes, what were your reasons for having oral, vaginal or anal sex?

Mark all that apply

I had a boyfriend / girlfriend	☐	I was forced to	☐
I felt pressured by my friends	☐	My boyfriend / girlfriend said if I loved them I should have sex with them	☐
I wanted to have sex	☐	My partner and I thought that sex would make us closer	☐
I wanted money, gifts	☐	Other<br>Specify _ _ _ _ _ _ _ _ _ _ _ _ _ _	☐

6.10. How old were you when you first had oral, vaginal or anal sex ? **Please write in a number**

Oral sex ☐☐ Years old

Vaginal sex ☐☐ Years old

Anal sex ☐☐ Years old

6.11. Think about each partner that you have had either oral, vaginal or anal sex with. If you add them up, about how many different sex partners have you ever had (including your current sex partner(s)) ?
Please write in a number

☐☐☐ Sex partners

6.12. How many of these partners were **new** partners in the last year? **Please write in a number**

☐☐☐ Sex partners

Version 1 . 0

Date ☐☐ dd ☐☐☐ MMM ☐☐ yy

Staff Initials ☐☐☐

CAPRISA 007 Plate 031

Visit Code 1 0 0 0

A

Participant ID

0 0 7 - [ ][ ] - [ ][ ][ ][ ]

Study Site Participant

Relationships

6.13.1 Think about the age of the sex partners that you have had, of these sex partner(s) what was the age of the **oldest one**? **Please write in a number**

[ ][ ] Years old

6.13.2 How old were you when you first had sex with this person ? **Please write in a number**

[ ][ ] Years old

6.13.3 Have you ever had the following types of sex with this sex partner **(oldest partner)**, and how often did you use a condom during these acts?

			How often did you use a condom in these acts ?			
**Type of Sex**	**Yes**	**No**	**Never**	**Some of the time**	**Most of the time**	**Always**
Oral Sex	☐	☐	☐	☐	☐	☐
Vaginal Sex	☐	☐	☐	☐	☐	☐
Anal Sex	☐	☐	☐	☐	☐	☐

6.14.1 Think about all the sex partners that you have had in the last 30 days, if you add them up, how many sex partners have you had in the last 30 days? **Please write in a number**

[ ][ ] Sex partners

OR

☐ I have not had sex in the last 30 days

If none, please skip to question 6.15.1

6.14.2 Of the sex partner(s) you have had in the last 30 days, how old was the **oldest one**? **Please write in a number**

[ ][ ] Years old

Version 1 . 0

Date [ ][ ] dd [ ][ ][ ] MMM [ ][ ] yy

Staff Initials [ ][ ][ ]

CAPRISA 007 Plate 032

Visit Code 1 0 0 0

A

Participant ID

0 0 7 - __ __ - __ __ __ __

Study Site Participant

Relationships

6.14.3 In total, about how many times have you had the following types of sex in the last 30 days and how often did you use a condom during these acts ?

		How often did you use a condom in these acts ?			
**Type of Sex**	**Number of times**	**Never**	**Some of the time**	**Most of the time**	**Always**
Oral sex	☐☐☐	☐	☐	☐	☐
Vaginal sex	☐☐☐	☐	☐	☐	☐
Anal sex	☐☐☐	☐	☐	☐	☐

6.15.1 Think about the age of the sex partner that you last had sex with, what was the age of this partner ?
Please write in a number

☐☐ Years old

6.15.2 How old where you when you first had sex with this person ?
Please write in a number

☐☐ Years old

6.15.3 The last time you had sex, what types of sex did you have. And did you use a condom during these acts ?

	**Yes / No**		**Did you use a condom ?**	
**Type of Sex**	**Yes**	**No**	**Yes**	**No**
Oral sex	☐	☐	☐	☐
Vaginal sex	☐	☐	☐	☐
Anal sex	☐	☐	☐	☐

Version 1.0

Date ☐☐ ☐☐☐ ☐☐ (dd MMM yy)

Staff Initials ☐☐☐

Participant ID

0 0 7 - _ _ - _ _ _ _

Study Site Participant

Relationships

6.16. Have any of the sex partners that you have ever had, had other sex partners at the same time as they were with you?

Yes ☐

No ☐

Do not know ☐

6.17. Have you ever had more than one sex partner at a time, where one partner **doesn't know** you are having sex with someone else?

Yes ☐ No ☐

6.18. Have you ever had more than one sex partner at a time, where one partner **did know** that you were having sex with someone else?

Yes ☐ No ☐

6.19. Have any of the sex partner(s) that you have had ever had a HIV test?

Yes ☐ → **If yes, please go to question 6.20**

No ☐ → **If no, please skip to question 6.21**

Do not know ☐ → **If you don't know, please skip to question 6.21**

6.20 If yes, are you aware of his / her HIV status ?

Yes ☐ No ☐

CAPRISA 007 Plate 034

Visit Code 1 0 0 0

F

Participant ID

0 0 7 - _ _ - _ _ _ _
Study Site Participant

Relationships

6.21 Of the sex partner(s) that you have had, how many have been circumcised ?

Mark only one

All of them	☐	Do not know how many have been circumcised	☐
Some of them	☐	I do not know what circumcision is	☐
None of them	☐		

6.22 Have you ever had sex with someone so that they would give you money, food, airtime or shelter , or for anything else that you did not have ?

Yes ☐

No ☐

6.23 I have had sex but have decided to stop having sex ?

Yes ☐ → **If yes, please go to question 6.24**

No ☐ → **If no, please go to question 7.1**

End of Section A: Thank you for answering these questions, please go to Question 7.1

CAPRISA 007 Plate 035

Visit Code 1 0 0 0

F

Participant ID

0 0 7 - _ _ - _ _ _ _
Study Site Participant

Relationships

SECTION B

If you have never had oral, vaginal or anal sex please answer the following questions:

6.24 If you have not had oral,vaginal or anal sex, why have you chosen not to ?

Mark all that apply

I do not have a boyfriend / girlfriend	☐	I am scared of getting an STI (Sexually Transmitted Infection)	☐
I want to wait for marriage	☐	I don't want to fall pregnant	☐
I have not had the chance to have sex	☐	Sex is something that is not important to me	☐
I want to stay a virgin	☐	I am scared of getting HIV	☐
Other<br>Specify_ _ _ _ _ _ _ _ _ _ _ _ _	☐		

6.25 Read each of the following statements about reasons for not having sex and say if you agree or disagree

Statement	Agree	Disagree
It's okay to **not** to have sex	☐	☐
Having a boyfriend/girlfriend does not have to involve sex	☐	☐
It is okay to still be a virgin when you are 18 years old	☐	☐
I feel pressured to have sex	☐	☐
It is important to finish school before you have sex	☐	☐
Having sex with an older partner could get you things like food, airtime or transport	☐	☐
I am scared of falling pregnant therefore I do not have sex	☐	☐
I don't feel that I need to prove my fertility to my partner	☐	☐
I am scared of getting HIV therefore I am not having sex	☐	☐
I don't want to have sex until I am married	☐	☐
I don't think I am old enough to have sex	☐	☐

Version 1.0

Date _ _ / _ _ _ / _ _
dd MMM yy

Staff Initials _ _ _

CAPRISA 007 Plate 036

Visit Code 1 0 0 0

F

Participant ID

0 0 7 - [ ][ ] - [ ][ ][ ][ ]

Study Site Participant

Menstruation, Pregnancy and Contraception

In this section we are going to ask you some questions about menstruation, pregnancy and contraception (Contraceptives are things people use to stop themselves falling pregnant)

First we would like to ask you about menstruation

7.1 How old were you when you had your first menstrual period?

[ ][ ] Years old

OR

☐ I have not had my menstrual period yet

If no, please skip to question 7.7

7.2 Do you get your menstrual period at about the same time each month ?

Yes ☐ No ☐

7.3 Have you ever missed your menstrual period ?

Yes ☐ No ☐ → **If no, please skip to question 7.5**

7.4 How often do you miss your menstrual period?

Never ☐

Sometimes ☐

Quite a few times ☐

Participant ID

0 0 7 - __ __ - __ __ __ __

Study Site Participant

Menstruation, Pregnancy and Contraception

7.5 How many days ago was your last menstrual period? **(Please write in a number)**

☐☐ Days

7.6 If more than 30 days ago, have you had a pregnancy test?

Yes ☐

No ☐

My last menstrual period was less than 30 days ago ☐

Now, we are going to ask you a few questions about pregnancy:

7.7. How important is it for you **not** to fall pregnant while you are still at school?

Very important ☐

Important ☐

Not important ☐

Participant ID

0 0 7 - [ ][ ] - [ ][ ][ ][ ]

Study Site Participant

Menstruation, Pregnancy and Contraception

7.8. Please read each of the following statements about pregnancy and say if you agree or disagree with them

Statement	Agree	Disagree
I feel that I am too young to fall pregnant	☐	☐
If a boyfriend will look after a young girl then it is ok for her to fall pregnant while she is at school	☐	☐
I do not want to be pregnant now because I cannot afford to have a baby	☐	☐
If I had a baby I would have to leave school	☐	☐
It is not the responsibility of the boy to look after the baby if he makes a girl pregnant	☐	☐
I need to finish school before I fall pregnant	☐	☐
I want to be a parent and do not mind falling pregnant at school	☐	☐
I am scared that I am going to die soon and not have a chance to have children	☐	☐
If my partner wanted me to have a baby, I would have one	☐	☐
It is okay to fall pregnant if you can access child support grants	☐	☐
I need to have a steady boyfriend before thinking about falling pregnant	☐	☐
My family would be extremely cross with me if I became pregnant	☐	☐

CAPRISA 007 Plate 039

Visit Code 1 0 0 0

F

Participant ID

0 0 7 - _ _ - _ _ _ _
Study Site Participant

Menstruation, Pregnancy and Contraception

7.9 Have you ever been pregnant ?

Yes ☐ ☐ No → **If no, please skip to question 7.18**

7.10 How old were you the **first time** you became pregnant ? **Please write in a number**

☐☐ Years old

7.11 At that time, when you first became pregnant, how old was the person who made you pregnant ?
Please write in a number

☐☐ Years old

7.12 How many times have you been pregnant ? **Please write in a number**

☐☐ Times

7.13 How many of these pregnancies were not planned (by accident) ? **Please write in a number**

☐☐ Times

7.14 How many children have you given birth to ? **Please write in a number**

☐☐ Children

7.15 How many of these children were alive at birth ? **Please write in a number**

☐☐ Children

Participant ID

0 0 7 - [ ][ ] - [ ][ ][ ][ ]

Study Site Participant

Menstruation, Pregnancy and Contraception

7.16 How many of your children are still alive now ? **Please write in a number**

[ ][ ] Children

7.17 Have you ever had an abortion ?

Yes ☐ ☐ No

If you have not been pregnant please answer the following questions:

7.18 What has stopped you from falling pregnant?

Mark all that apply

I do not have a boyfriend	☐	I use a family planning method	☐
I think I am too young to fall pregnant	☐	My partner and I use a condom when having sex	☐
I want to finish school before I fall pregnant	☐	I am still a virgin	☐
Other<br>Specify _ _ _ _ _ _ _ _ _ _ _ _ _ _	☐		

7.19 At what age would you like to start having children ? **Please write in a number**

[ ][ ] Years old

CAPRISA 007 Plate 041

Visit Code 1 0 0 0

F

Participant ID

0 0 7 - ☐☐ - ☐☐☐☐

Study Site Participant

Menstruation, Pregnancy and Contraception

Now we would like to ask you about contraception. Contraception is things people use to avoid getting pregnant (also called family planning).

7.20. Which of the following methods of contraception (methods to prevent pregnancy) did you know about before today? (Mark all the ones you knew about below)

☐	Birth Control Pills	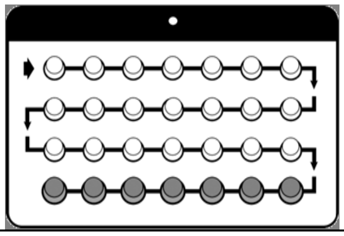	☐	Rhythm or calendar or "safe period" method	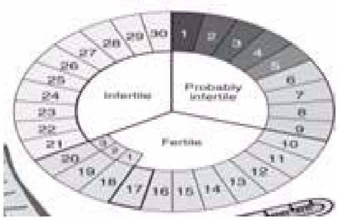
☐	Injectable contraceptives e.g. Nuristerate or Depo-Provera	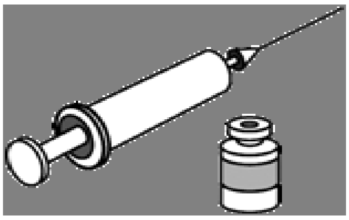	☐	Implants	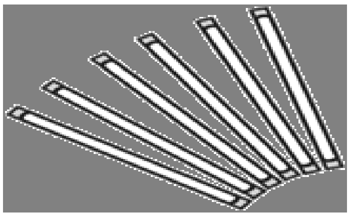
☐	Female condoms	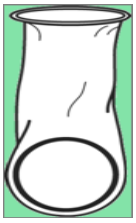	☐	Vasectomy (Male Sterilization)	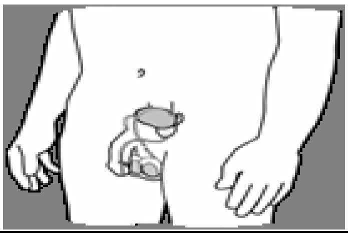
☐	Diaphragm	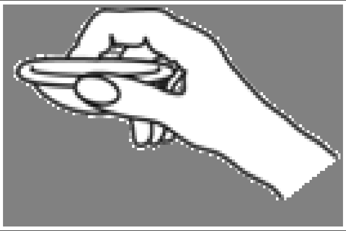	☐	Male condoms	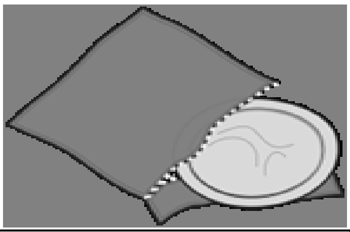
☐	Intrauterine device (IUD)	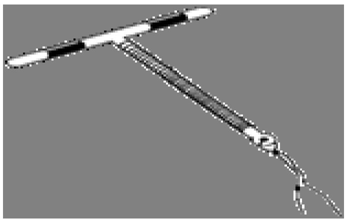	☐	Emergency contraception	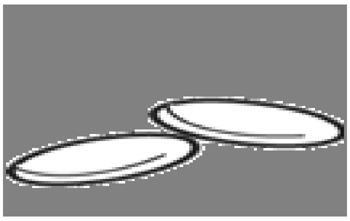
☐	Spermicide	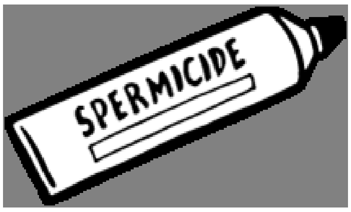	☐	Tubal Ligation (Female Sterilization)	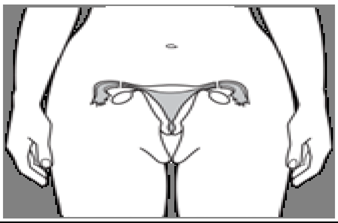

Version 1.0

Date ☐☐ ☐☐☐ ☐☐
dd *MMM* *yy*

Staff Initials ☐☐☐

CAPRISA 007 **Plate 042**

Visit Code 1 0 0 0

F

Participant ID

0 0 7 - ☐☐ - ☐☐☐☐

Study Site Participant

Menstruation, Pregnancy and Contraception

7.20 Continued ... Which of the following methods of contraception (methods to prevent pregnancy) did you know about before today? **(Mark all the ones you knew about below)**

☐	Withdrawal Method	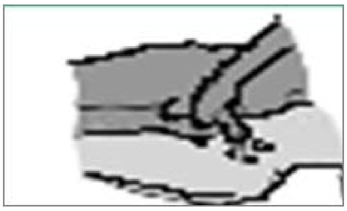	☐	Masturbation	Ways that people seek out and/or experience *sexual* pleasure by themselves, without a sexual partner.
☐	Thigh Sex	A man has sexual intercourse with his partner through their clamped thighs.	☐	Other: Specify	_ _ _ _ _ _ _ _ _ _<br>_ _ _ _ _ _ _ _ _ _

7.21. Of the following contraceptive methods, please tell us how well you think each one protects against pregnancy?

	**Not Protective**	**Protective**	**Do not know**
Birth control pills	☐	☐	☐
Injectable contraceptives e.g. Nuristerate or Depo-Provera	☐	☐	☐
Female condoms	☐	☐	☐
Male condoms	☐	☐	☐
Diaphragm	☐	☐	☐
Intrauterine device (IUD)	☐	☐	☐
Spermicide	☐	☐	☐
Tubal ligation (Female sterilization)	☐	☐	☐
Withdrawal method	☐	☐	☐
Rhythm or calender or “safe period” method	☐	☐	☐
Vasectomy (Male sterilization)	☐	☐	☐
Implants	☐	☐	☐
Emergency contraception	☐	☐	☐
Thigh Sex	☐	☐	☐
Masturbation	☐	☐	☐

Menstruation, Pregnancy and Contraception

7.22 Where would you get contraceptives if you needed them ?

Mark all that apply

At the clinic close to my school or home	☐	At a clinic **not** close to my school or home	☐
At the hospital	☐	At school	☐
I do not know	☐	Other<br>Specify_ _ _ _ _ _ _ _ _ _ _ _ _ _ _	☐

7.23 Whose responsibility is it to make sure you have contraception ?

Mark one only

Female partner	☐	Male partner	☐
Both partners	☐	Contraception is not important	☐
Other<br>Specify _ _ _ _ _ _ _ _ _ _ _ _ _ _	☐		

If you have had sex before, please answer questions 7.24 to 7.27

If you have not had sex before, please go to question 8.1

7.24. Have you or your partner ever used contraception?

Yes ☐ ☐ No → **If no, please skip to question 8.1**

7.25. Are you or your partner currently using any contraception?

Yes ☐ ☐ No

Participant ID

0 0 7 - _ _ - _ _ _ _

Study Site Participant

Menstruation, Pregnancy and Contraception

7.26. What types of contraception are you or your partner currently using or have used before?
(Mark all that apply)

Birth control pills	☐
Injectable contraceptives e.g. Nuristerate or Depo-Provera	☐
Female condoms	☐
Male condoms	☐
Diaphragm	☐
Intrauterine device (IUD)	☐
Spermicide	☐
Withdrawal method	☐
Rhythm or calender or “safe period” method	☐
Implants	☐
Emergency contraception	☐
Other: Specify _	☐

7.27 Where do you get your contraceptive from ?

Mark all that apply

At the clinic close to my school or home	☐	At a clinic **not** close to my school or home	☐
At the hospital	☐	At school	☐
Other<br>Specify _ _ _ _ _ _ _ _ _ _ _ _ _	☐		

CAPRISA 007 Plate 045

Visit Code 1 0 0 0

F

Participant ID

0 0 7 - ☐☐ - ☐☐☐☐

Study Site Participant

Health Screening

We would like to ask you a few questions about different aspects of your health. We will ask you some questions about sexually transmitted infections and about TB.

Sexually transmitted infections (STI)	A term used to describe diseases that are transmitted through the exchange of semen, blood, and other body fluids; or by direct contact with part of the body of another person that has a STI.

8.1. Have you ever had any the following symptoms?

	Yes	No
Vaginal discharge/ Urethral discharge	☐	☐
Genital Sores/Ulcers	☐	☐
Burning Pain on Urination	☐	☐

If you have had any of the three symptoms, please go to question 8.2

If you have never had any of the three symptoms, please skip to question 8.7

8.2. Do you currently have any of the following symptoms?

	Yes	No
Vaginal discharge/ Urethral discharge	☐	☐
Genital Sores/Ulcers	☐	☐
Burning Pain on Urination	☐	☐

8.3. If you have ever had, or currently have, any of these symptoms, did you tell your boyfriend/girlfriend that you had these symptoms?

Yes ☐ No ☐

Participant ID

0 0 7 - __ __ - __ __ __ __
Study Site Participant

Health Screening

8.4. If you have ever had, or currently have, any of these symptoms, did you get treatment for these symptoms?

	Yes	No
Vaginal discharge/ Urethral discharge	☐	☐
Genital Sores/Ulcers	☐	☐
Burning Pain on Urination	☐	☐

If you did get treatment for any of the symptoms, please go to question 8.5

If you never got treatment for any of the symptoms, please skip to question 8.7

8.5. If you did get treatment for any of the symptoms, where did you go? **Mark all that apply**

Clinic	☐	Hospital	☐
Traditional Healer	☐	Other<br>Specify_ _ _ _ _ _ _ _ _ _ _ _	☐

8.6. Did you inform your partner that you had treatment?

Yes ☐ No ☐

TB Questions

8.7 Please mark if you have any of the following symptoms, and if yes, how long you have had them?

Symptom	Yes	No	If yes, how long have you had it ?	
			More than 2 weeks	2 weeks or less
I have had a cough	☐	☐	☐	☐
Loss of appetite	☐	☐	☐	☐
Unexplained weight loss	☐	☐	☐	☐
Drenching night sweats	☐	☐	☐	☐
Fevers and shivering at the same time	☐	☐	☐	☐

Date __ __ / __ __ __ / __ __
dd MMM yy

Staff Initials __ __ __

CAPRISA 007 Plate 047

Visit Code 1 0 0 0

A

Participant ID

0 0 7 - ☐☐ - ☐☐☐☐

Study Site Participant

Substance Use

We are now going to talk to you about recreational drugs, please look at the questions below and answer as honestly as possible

9.1 Have you ever used any of the following substances and how often have you used them ?

Substances (common name)	Everyday	Three or more times a week	Once weekly	Once a month or less	Never
**Used cigarettes**	☐	☐	☐	☐	☐
**Drank alcohol**	☐	☐	☐	☐	☐
**Dagga**	☐	☐	☐	☐	☐
**Benzodiazepine** (benzos / valium / pinkies / tranks / date-rape drug)	☐	☐	☐	☐	☐
**Cocaine** (coke / snow / flake / blow / candy)	☐	☐	☐	☐	☐
**Codeine** (painkillers)	☐	☐	☐	☐	☐
**Crack** (rock)	☐	☐	☐	☐	☐
**Ecstacy** (doves / Adam & Eve / fido-dido / snowballs / apples / domes / MDMA)	☐	☐	☐	☐	☐
**GHB** (G / liquid X / scoop / soap / grievous bodily harm / gook)	☐	☐	☐	☐	☐
**Heroin** (smack / ska / H / junk / sugars)	☐	☐	☐	☐	☐
**Solvents** (petrol / whipets / poppers / snappers)	☐	☐	☐	☐	☐
**LSD** (acid / candy / caps / microdots / sunshine / smarties)	☐	☐	☐	☐	☐
**Mandrax** (mandies / mx / buttons / whites)	☐	☐	☐	☐	☐
**Amphetamines** (speed / meth / chalk / glass / ice / tik / dexies / crank)	☐	☐	☐	☐	☐
**Opium** (skee / joy plant / pen yan)	☐	☐	☐	☐	☐
**Other : Specify** _ _ _ _ _ _ _ _ _ _ _	☐	☐	☐	☐	☐

9.2 Have you ever injected any of these drugs ?

Yes ☐ No ☐ → **If no, please go to question 10.1**

9.3 How often do you inject these drugs ?

Mark only one

Everyday	☐	Once weekly	☐
Two or more times a week	☐	Once a month or less	☐

Version 1 . 0

Date ☐☐ ☐☐☐ ☐☐ (dd MMM yy)

Staff Initials ☐☐☐

CAPRISA 007 Plate 048

Visit Code 1 0 0 0

A

Participant ID

0 0 7 - ☐☐ - ☐☐☐☐

Study Site Participant

After school/Extra-mural activities

Finally, we would like to ask you some questions about your afterschool/extra-mural activities.

10.1 What after school/extramural activities are you currently engaged in ?

Mark all that apply

Sport	☐	Cultural activities (for example debating, chess, public speaking)	☐
Community work	☐	Traditional activities (for example Stick fighting, Zulu dancing, Drama)	☐
Social clubs	☐	Church activities	☐
None	☐	My school does not offer after school / extramural activities	☐
Other : Specify _ _ _ _ _ _ _ _ _ _	☐		

10.2 How important is participating in after school / extra-mural activities to you ? **Mark only one**

Very important ☐

Important ☐

Not important ☐

10.3 About how much time do you spend doing after school/extramural activities each day ?
Please write in numbers

Monday	Tuesday	Wednesday	Thursday	Friday	Saturday	Sunday
☐☐ hrs	☐☐ hrs	☐☐ hrs	☐☐ hrs	☐☐ hrs	☐☐ hrs	☐☐ hrs

10.4 About how much time do you spend doing homework each day ?
Please write in numbers

Monday	Tuesday	Wednesday	Thursday	Friday	Saturday	Sunday
☐☐ hrs	☐☐ hrs	☐☐ hrs	☐☐ hrs	☐☐ hrs	☐☐ hrs	☐☐ hrs

Version 1 . 0

Date ☐☐ ☐☐☐ ☐☐
dd *MMM* *yy*

Staff Initials ☐☐☐

CAPRISA 007 Plate 049

Visit Code 1 0 0 0

A

Participant ID 0 0 7 - _ _ - _ _ _ _
Study Site Participant

After school/Extra-mural activities

10.5 Why **do** you take part in your after school / extra-mural activity ? **Mark all that apply**

It is fun	☐	I like the team work involved	☐
It is fun to be competitive	☐	I get to see my friends	☐
It teaches me the importance of discipline and commitment	☐	It makes me fit and strong	☐
It teaches me the importance of working with other people	☐	It allows me to contribute to my community	☐
I do not take part in extramural/after school activities	☐	Other Specify_ _ _ _ _ _ _ _ _ _ _ _ _ _	☐

10.6 Why **don't** you take part in any after school / extra-mural activity ?
Mark all that apply

I have not found an activity I enjoy	☐	I am shy	☐
I am too busy with household chores	☐	I do not like working in a team	☐
My school has no cultural activities	☐	I do not like sport	☐
Other : Specify _ _ _ _ _ _ _ _ _ _	☐		

10.7 When you're with your friends outside of school, what do you usually do? **Mark all that apply**

We spend time at each other's houses	☐	We play sports together	☐
We go to the tavern	☐	Sit around and talk	☐
Other : Specify _ _ _ _ _ _ _ _ _ _	☐		

10.8 My friends that I spend the most time with are : **Mark only one**

Mostly boys ☐

Mostly girls ☐

Boys and girls ☐

CAPRISA 007 Plate 050

Visit Code 1 0 0 0

F

Participant ID 0 0 7 - _ _ - _ _ _ _
Study Site Participant

After school/Extra-mural activities

10.9 If you were rewarded for doing well in school, getting an HIV test or participating in extra-curricular activities what type of reward would you prefer.

Mark all that apply

Money	☐	Mall voucher	☐
Clothes	☐	Cosmetics	☐
School fees paid	☐	Money in an account	☐
School uniform	☐	Airtime	☐
Other : Specify: _ _ _ _ _ _ _ _ _ _ _ _	☐		

Review Questions

	**We would like to review some answers to certain questions we have asked you**	**Yes**	**No**
7.5	Has it been more than 30 days since you last menstruated	☐	☐
8.1	Are you currently experiencing vaginal discharge/urethral discharge/genital sores or ulcers, or a burning pain during urination	☐	☐
8.7	Do you have any of the following symptoms: (a cough, loss of appetite, unexplained weight loss, drenching night sweats, fevers and shivering at the same time as the fever) for more than 2 weeks	☐	☐
	If you answered yes to any of these questions, we would like to encourage you to speak to one of the CAPRISA staff here today about any questions you may have about these symptoms, and do a quick check on your health as a free service to you. If you have answered no, please note that a staff member is still available to talk to you about your health.		

We would like to thank you for participating in this study. Please know that all information that you write on these questionnaires will be stored safely and not shared with others and that any reports that are made as part of the study will not identify you. If you would like to discuss anything about this questionnaire, or would like to discuss anything else, please feel free to speak to us. You can contact us using the telephone numbers that you find at the end of your informed consent document. Thank you.

Version 1 . 0

Date _ _ / _ _ _ / _ _
dd MMM yy

Staff Initials _ _ _
